# Synthesis and biological evaluation of the progenitor of a new class of cephalosporin analogues, with a particular focus on structure-based computational analysis

**DOI:** 10.1371/journal.pone.0181563

**Published:** 2017-07-27

**Authors:** Anna Verdino, Giovanni Vigliotta, Deborah Giordano, Ivana Caputo, Annunziata Soriente, Margherita De Rosa, Anna Marabotti

**Affiliations:** Department of Chemistry and Biology "A. Zambelli", University of Salerno, Fisciano (SA), Italy; Emory University School of Medicine, UNITED STATES

## Abstract

We present the synthesis and biological evaluation of the prototype of a new class of cephalosporins, containing an additional isolated beta lactam ring with two phenyl substituents. This new compound is effective against Gram positive microorganisms, with a potency similar to that of ceftriaxone, a cephalosporin widely used in clinics and taken as a reference, and with no cytotoxicity against two different human cell lines, even at a concentration much higher than the minimal inhibitory concentration tested. Additionally, a deep computational analysis has been conducted with the aim of understanding the contribution of its moieties to the binding energy towards several penicillin-binding proteins from both Gram positive and Gram negative bacteria. All these results will help us developing derivatives of this compound with improved chemical and biological properties, such as a broader spectrum of action and/or an increased affinity towards their molecular targets.

## Introduction

Beta lactam antibiotics are considered among the most important drugs for the history of mankind, and, due to their ease of delivery, potent activity, relatively low toxicity and low costs, they still remain among the most frequently used classes of antimicrobial drugs [1]. Their targets are the penicillin-binding proteins (PBPs), a large family of enzymes not present in eukaryotes, which are involved in the synthesis and maintenance of the peptidoglycan, the main component of the bacterial cell wall. All PBPs share a common DD-peptidase activity that could be either a DD-transpeptidase, a DD-carboxypeptidase or a DD-endopeptidase activity, catalyzed by a common domain (the so-called penicillin-binding (PB) domain), made of two subdomains with the active site lying at the interface between them. The reactions catalyzed by the PB domain follow a three-step mechanism with the formation of an acyl-enzyme covalent intermediate through a Ser residue conserved in all the PBPs. There are many classes of these enzymes, depending on their molecular mass, structure and different additional activities held by other domains possibly present in their structures, and there are many different PBPs in each bacterial strain [2,3].

The beta lactam antibiotics resemble to the natural substrate of PBPs (the D-Ala-D-Ala dipeptide that ends the pentapeptide precursors of the peptidoglycan and that forms the cross-links between the glycan strands of the peptidoglycan), thus they act as suicide inhibitors for these enzymes. Indeed, the catalytic Ser residue attacks the carbonyl of the beta lactam ring, with the formation of a covalent acyl-enzyme complex that is hydrolyzed at a very low rate, therefore preventing further reactions. Blocking enzyme activity has a lethal effect because it produces the inhibition of the growth of the bacteria, or their lysis via several mechanisms that involve a global imbalance of the cell wall metabolism [4].

Since their discovery, many different classes of beta lactam antibiotics have been developed, first in order to broaden their spectrum of actions or to improve some pharmacological features, then to overcome the increasing bacterial resistance towards the original molecules [5,6]. The most common way by which bacteria defend themselves from beta lactam antibiotics is the synthesis of beta lactamases, hydrolytic enzymes able to cleave the beta lactam ring thus destroying its antibacterial properties. In fact, the intact beta lactam core is essential either to mimic the structure of the D-Ala-D-Ala dipeptide, thereby facilitating the binding to the active site of PBPs, and to increase the reactivity of these molecules towards linear amides, allowing them to compete efficiently with the natural substrate for the reaction of acylation. The first beta lactamase enzymes have been isolated few years after the discovery of penicillin. Since then, an impressively increasing number of unique beta lactamase enzymes has been identified and by now they are widespread mainly because of horizontal gene transfer phenomena in the microbial communities under the selective pressure deriving from the use and misuse of antibiotics in human applications [7].

Another strategy exploited by bacteria to escape from the killing action of beta lactams is the expression of PBPs with reduced affinity with respect to these antibiotics. The first example in this sense has been the PBP2a expressed by the first strain of methicillin-resistant *S*. *aureus* isolated after the introduction of methicillin in the therapy [8]. This enzyme has a very low affinity for beta lactam antibiotics, and a very low acylation rate: these two factors work in concert preventing the enzyme acylation by the antibiotics *in* vivo [9]. Also mutations in the PBPs can lead to enzymes with decreased affinity towards these molecules [4].

In the recent decades, a continuous increase of the resistance against antimicrobial drugs has been registered and is becoming a global concern. Dr. Keiji Fukuda, Assistant Director-General for the Health Security, stated in its introduction to the WHO’s 2014 report on global surveillance of antimicrobial resistance: “A post-antibiotic era—in which common infections and minor injuries can kill—far from being an apocalyptic fantasy, is instead a very real possibility for the 21st century” [10]. Indeed, the global development of bacterial resistance is seriously impairing our ability to cure even the more common infectious diseases. In particular, from the surveillance made by WHO, it appears that many bacteria, especially the so-called ESKAPE pathogens [11] are becoming more and more resistant to different antibiotics, in such way for many patients infected by these bacterial strains there are currently no effective treatments [10]. Therefore, it is essential to continue developing new antibiotics in order to overcome this problem. Unfortunately, the last completely new class of antibiotics was discovered about 30 years ago [12]. Thus, while the efforts to find new antibacterial drugs proceed [13,14], it is essential to develop new molecules belonging to the existing classes of antibiotics, trying to introduce innovative solutions in order to bypass the bacterial defenses against them.

As a part of our ongoing studies on finding new potential antibiotics [15,16], we have recently synthesized a set of new 6-aminopenicillanic acid (6-APA) derivatives, containing an additional beta lactam ring with different substituents, joined to the amino-nitrogen of 6-APA scaffold *via* an amide bond.We have shown that the compounds obtained in that way exhibited antibacterial activity against Gram positive bacteria (microbial inhibitory concentration (MIC) in the micromolar range), with no or minimal *in vitro* cytotoxicity at a concentration comparable or higher than the MICs found in examined Gram positive bacteria [17]. In the present work, we have extended this approach to the 7-aminocephalosporanic (7-ACA) acid, and we have synthesized the prototype of a new class of cephalosporin analogues, testing it for its efficacy towards both Gram positive and negative bacteria, and its safety towards eukaryotic cell lines. Moreover, in an effort to rationalize the creation of the most effective derivatives of this new lead compound, we have applied a computer-aided analysis using covalent docking. This innovative strategy allows evaluating the interaction of a compound covalently bound to a target, such as in the case of beta lactam antibiotics, to dissect the interactions between that molecule and some selected PBPs, representative of different class of these enzymes from different bacteria. The results have been compared with those obtained with ceftriaxone, a third-generation cephalosporin with an *in vitro* activity against Gram positive and Gram negative aerobic and anaerobic bacteria, widely used in clinics to treat microorganisms that tend to be resistant to many other antibiotics. The information obtained in this way will allow us to develop new molecules belonging to this class of compounds, with even improved features, compared to this lead compound.

## Results

### Chemistry

**Fig 1:**
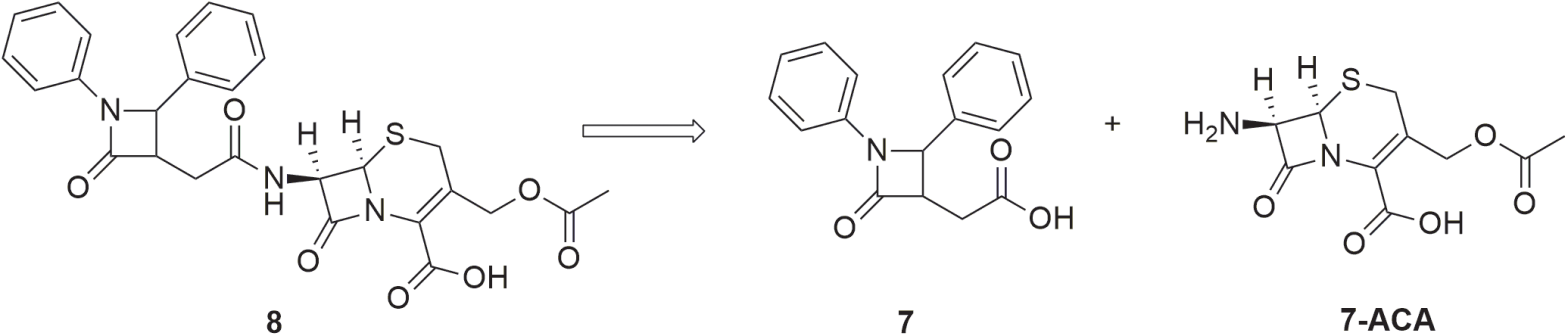
Retrosynthetic strategy for title compound 8.

The cephalosporin analogue **8** was planned to result from a simple strategy based on the coupling of 7-ACA with a functionalized 2-azetidinone ring built through [2+2] cycloaddition reaction of a ketene with an imine (Staudinger reaction) as illustrated in Fig 1. Recently, we used a similar strategy for the synthesis of new penicillin-type analogues, displaying its synthetic potentialities [17].

**Fig 2:**
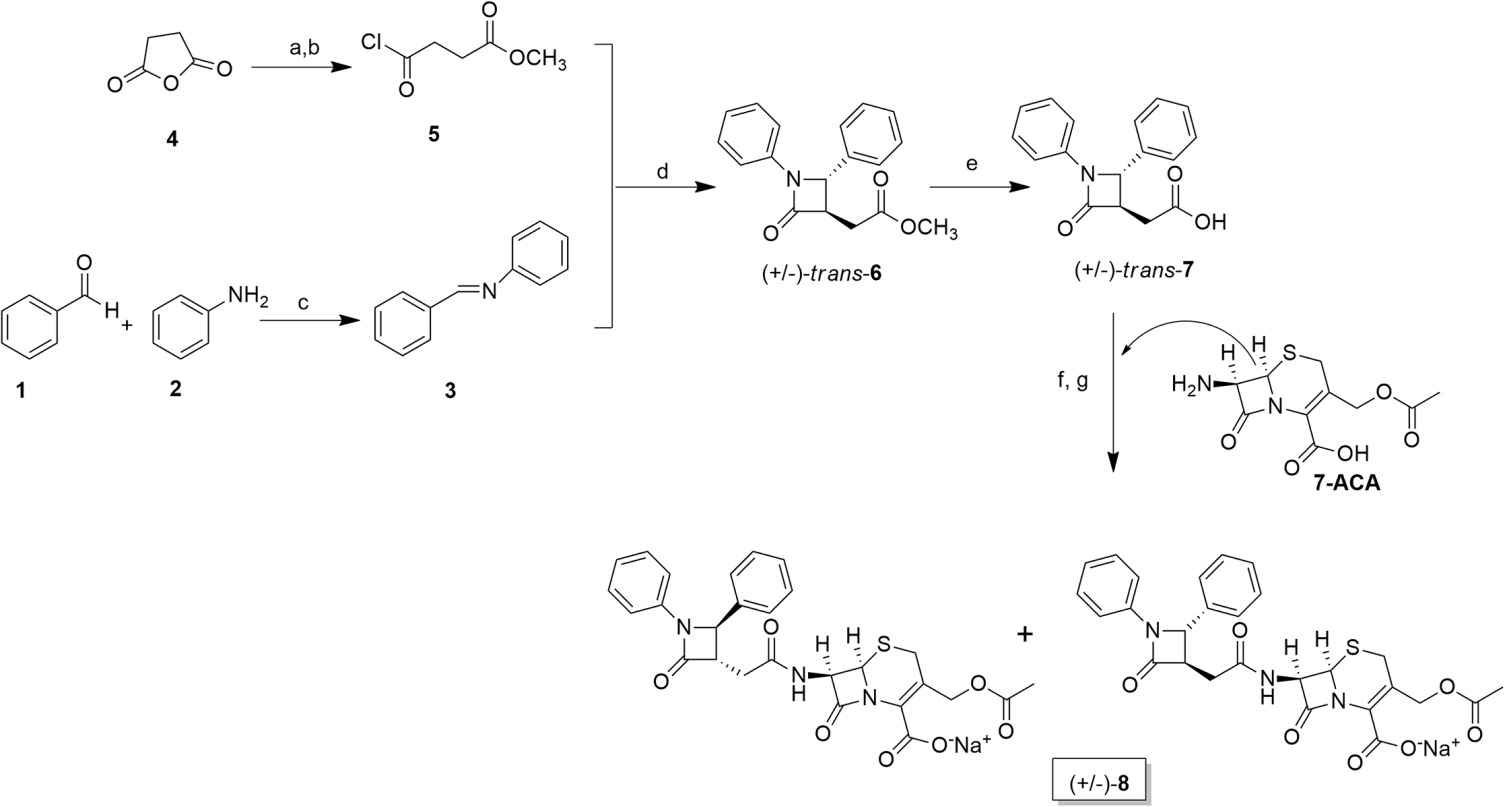
Reagents and conditions for the synthesis of compound 8. (a) MeOH, reflux; (b) SOCl_2_, reflux; (c) EtOH, reflux; (d) *n*Bu_3_N, toluene, reflux; (e) LiOH, THF/H_2_O; (f) DCC, Et_3_N, CH_2_Cl_2_; (g) CH_3_COCH_3_, NaHCO_3_, H_2_O.

Synthesis started with the Staudinger reaction [18–21] between imine **3** and ketene obtained *in situ* from the corresponding acid chloride **5** under basic conditions, to give the racemic compound (+/-)-*trans*-**6** (Fig 2). The control of the experimental reaction conditions such as solvent choice, temperature and order of addition of the reagents, governed the stereochemical outcome of the reaction affording only *trans* cycloadduct in moderate yield (60%). The assignment of the stereochemistry was made by ^1^H-NMR spectra measuring the ^3^J coupling constants between H-3 and H-4 and comparing them with literature data [19].

Subsequently, treatment of (+/-)-*trans*-**6** with LiOH provided the corresponding acid (+/-)-*trans*-**7** in high yield. Standard amide coupling with commercially available 7-ACA followed by transformation into sodium salt provided final target **8** after crystallization such as an inseparable mixture of two diastereomers (dr = 7:3). Since our efforts to separate these diastereomers were unsuccessful and the computational analysis showed no significant differences of binding affinities to all PBPs for both two diastereomers (see below), we decided to perform the biological assays on the diastereomeric mixture of **8**.

In addition, a 1D- and 2D-NMR study with the aim to assign the signals of the two diastereomers of the final compound **8** was performed (see S1–S4 Figs) and the diastereoisomeric ratio was determined by integration of ^1^H NMR signals attributed to diastereomers. The most typical features are summarized here. The ^1^H NMR spectrum (S1 Fig) revealed the presence of two distinct peaks closed to each other at 2.09 and 2.08 ppm attributable to methyl groups H-12 of two diastereomers, and two signals at 3.48 and 3.43 for H-3’ of the azetidinone additional ring. Close inspection of the 5.0–6.0 ppm region of the COSY spectrum of **8** in S2A Fig revealed the presence of two cross-peaks at 5.62/5.08 and 5.62/5.03 attributable to the coupling between H-7 and H-6 hydrogen atoms of the two diastereoisomers of **8**. In addition, close inspection of the 2.0–5.0 ppm region of the 2D COSY spectrum in S2B and S2C Fig allowed us to attribute the H3’-H4’-H5’ spin systems of the two diastereoisomers of **8**. In details, we revealed the presence of a cross-peak at 3.43/5.05 ppm between H-3' (major diastereisomer) and H-4' (major diastereoisomer) (S2B Fig) and finally a coupling with the signals at 2.90 and 2.95 ppm attributable to H-5' (major diastereoisomer) (S2C Fig). Interestingly, hydrogen atom H-3' attributable to minor diastereoisomer at 3.48 ppm showed in the 2D COSY a coupling with the signal at 5.00 ppm attributable to H-4' (S2B Fig). Finally, H-3’ (minor diastereoisomer) at 3.48 ppm showed in 2D COSY spectrum two cross-peaks with the signals attributable to methylene protons H-5' at 2.99 and 2.96 ppm (S2C Fig). The ^13^C-and HMQC spectra (S3 and S4 Figs, respectively) confirmed the assignments for the two diastereoisomers. Finally, by integration of the ^1^H NMR signals at 3.48 and 3.43 ppm attributable to H-3' protons of the two diastereoisomers of **8** we were able to measure a diastereoisomeric ratio of 70:30.

### Antimicrobial activity

The antibacterial activity of compound **8** was evaluated by determining the MIC and minimum lethal dose (MLD) according to the CLSI (Clinical and Laboratory Standards Institute, formerly the NCCLS) guidelines [17,22].

The compound was tested against both Gram positive (*Staphylococcus aureus*, *Bacillus* sp.) and Gram negative bacteria (*Escherichia coli*, *Salmonella enterica* serovar *Typhimurium*). As reference compound was used ceftriaxone, a third-generation cephalosporin, with antimicrobial activity against many Gram-negative and most Gram positive bacteria. Results are shown in Table 1.

**Table 1:** MIC and MLD (in μg/mL) obtained for compound 8 in comparison with ceftriaxone.

Compounds	Antibacterial activity	Microorganisms			
		Gram positive		Gram negative	
		*S*. *aureus*	*Bacillus sp*.	*E*. *coli*	*Salmonella*
**compound 8**	MIC_50_	0.75	<0.25	>256	>256
	MIC_100_	2.5	2.5	>256	>256
	MLD	>3	>2.5	>256	>256
**Ceftriaxone**	MIC_50_	>2; <4	<0.25	<0.25	<0.25
	MIC_100_	5.5	0.75	0.25	0.25
	MLD	>7	≥1	>1	≥2

Compound **8** did not exhibit sufficient antimicrobial activity towards the tested Gram negative bacteria (MIC_100_> 256 μg/mL). In contrast, it was very effective against Gram positive bacteria, as evidenced by low values of the MICs and MLDs (MIC_100_ = 2.5 μg/mL). In particular, MIC_100_ of compound **8** for *Staphylococcus aureus* was more than twice lower than that of the ceftriaxone (2.5 μg/mL and 5.5 μg/mL, respectively).

### Cytotoxicity assay on human cells

**Fig 3:**
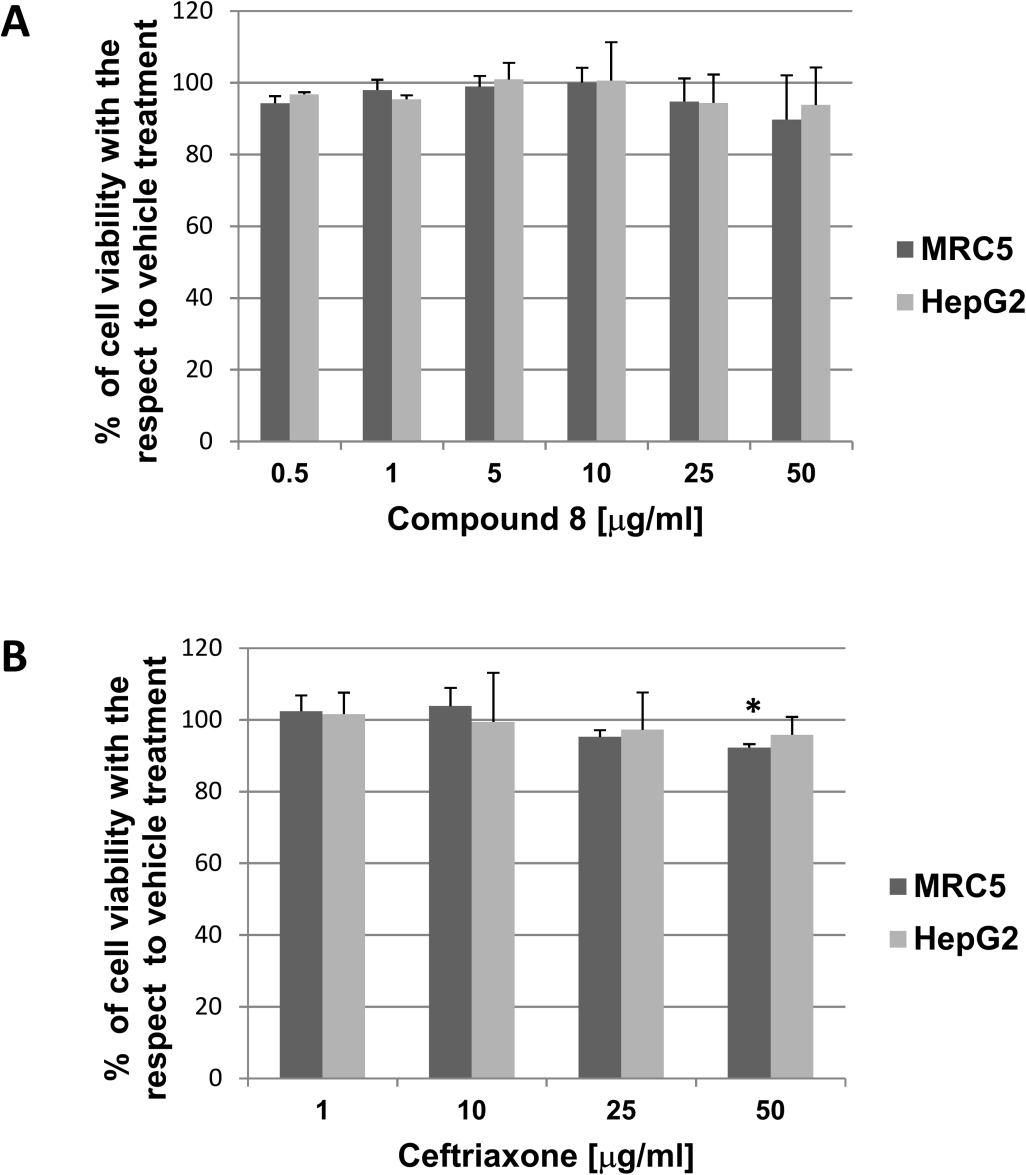
**Effect on cell viability of MRC5 and HepG2 cells after 24 h exposure to compound 8 (dose-range 0.5–50 μg/mL) (A) or ceftriaxone (dose-range 1–50 μg/mL) (B).** Data are reported as mean ± SD from two or three independent experiments each in triplicate. Vehicle (DMSO), at the highest concentrations used (0.2 and 0.4% v/v), induced a significant reduction of cell viability of 20% and 12% in MRC5 and HepG2 cells, respectively (not shown). *p<0.05 vs. the respective vehicle.

We performed the 3-(4,5-dimethylthiazol-2-yl)-2,5-diphenyltetrazolium (MTT) assay to investigate cytotoxicity of compound **8** on two different human cell lines, HepG2 and MRC5.We tested a panel of compound **8** concentrations from 0.5 μg/mL to 50 μg/mL to cover a wide range around measured MICs. We treated cells for 24 h in the presence of the compound, and then we evaluated residual cell viability, expressed as percent of viability obtained in the presence of vehicle only (dimethylsulfoxide (DMSO), at different concentration). We found that both MRC5 and HepG2 cells well tolerated compound **8** at almost all concentrations tested; indeed, we did not register any significant reduction of cell viability in the range 0.5–50 μg/mL (Fig 3A). Parallel experiments were conducted to test cytotoxicity of ceftriaxone (dose-range 1.0–50 μg/mL). We observed a slight (about 10%) but significant viability reduction at 50 μg/mL in MRC5 cells only (Fig 3B). As expected, the cytotoxic agent H_2_O_2_, used as positive control, decreased cell viability by over 90% (not shown).

### Computational analysis

We applied a covalent docking approach [23] in order to predict the interactions between the cephalosporin analogue compound **8** and selected PBPs [24–33] (Table 2), also in the view of obtaining useful information to modify the current compound in the future starting from a rational strategy.

**Table 2:** List of the structures of PBPs selected for the computational analysis.

Name	Class^a^	Organism	PDB file	Reference
**PBPs**				
PBP1b	A (HMM)	*E*. *coli*	5FGZ	[24]
PBP3	B (HMM)	*E*. *coli*	4BJP	[25]
**PBP4**	**C (LMM)**	***E*. *coli***	**2EX8**	**[26]**
PBP5	C (LMM)	*E*. *coli*	1Z6F	[27]
**PBP3**	**B (HMM)**	***P*. *aeruginosa***	**3OCL**	**[28]**
PBP5	C (LMM)	*P*. *aeruginosa*	4K91	[29]
PBP2a	B (HMM)	*S*. *aureus*	4CJN	[30]
**PBP3**	**B (HMM)**	***S*. *aureus***	**3VSL**	**[31]**
PBP4	C (LMM)	*S*. *aureus*	1TVF	[32]
PBP4a	C (LMM)	*B*. *subtilis*	2J9P	[33]

^a^: according to the classification reported in [3]. The proteins in bold were used to perform the self-docking test before running the covalent docking study with compound **8** and ceftriaxone.

First, we tested the reliability of this approach on our systems by using three complexes between selected PBPs and their co-crystallized ligands. Results are reported in S1 Table and in S5 Fig. It is possible to note that in general the covalent docking strategy has been able to reproduce the starting crystallographic complex, the best result being the one associated to 2EX8 (*E*. *coli* PBP4 with penicillin G).

Tables 3 and 4 report the results obtained for the docking of compound **8** to the selected proteins from Gram positive and negative bacteria, respectively. Our cephalosporin derivative is predicted to bind to all PBPs with a comparable binding energy, which is similar or even better than that of known antibiotics such as penicillin G, carbenicillin, cefotaxime (see also S1 Table) and ceftriaxone. In particular, it is possible to note that the results obtained for this last molecule are often more "scattered", with a consistent higher number of clusters found, and a higher incertitude for the identification of the best result (the representative pose with the best energy often is not the one corresponding to the cluster with the highest number of poses). This difference might indicate that ceftriaxone forms less stable complexes with the selected proteins with respect to compound **8**. The proteins showing an average better binding energy for compound **8** are PBP3 from *P*. *aeruginosa* and *S*. *aureus*, which are also the ones with the best predicted binding energy for ceftriaxone. From the predicted binding energies obtained by the docking results, it appears that all the PBPs tested, except PBP4 from *S*. *aureus*, bind preferably to the beta lactam ring of the 7-ACA moiety of compound **8**, with respect to the additional beta lactam ring. This is not equally evident for the PBP4 mentioned above, in which there is an inverse tendency, although the differences in predicted binding energies are not significant. Instead, no significant differences are present among the predicted binding energies of the two diastereoisomers in all classes of proteins.

**Table 3:** Results of covalent docking between beta lactam antibiotics and selected PBPs of Gram positive bacteria.

	4CJN–chain B (PBP2a from *S*. *aureus*)		3VSL (PBP3 from *S*. *aureus*)		1TVF (PBP4 from *S*. *aureus*)		2J9P (PBP4a from *B*. *subtilis*)	
Ligand	Predicted ΔG (kcal/mol) and number of poses in the selected cluster^a^	Total number of clusters	Predicted ΔG (kcal/mol) and number of poses in the selected cluster^a^	Total number of clusters	Predicted ΔG (kcal/mol) and number of poses in the selected cluster^a^	Total number of clusters	Predicted ΔG (kcal/mol) and number of poses in the selected cluster^a^	Total number of clusters
compound **8** (3R,4S), ring A reactive^b^	-1.06 (29)	13	-12.77 (11)	13	-12.71 (77)	7	-10.27 (33)	7
			-11.82 (41)				-9.48 (37)	
compound **8** (3R,4S), ring B reactive^c^	-4.49 (22)	16	-13.88 (28)	17	-11.94 (4)	15	-12.05 (57)	12
	-1.96 (31)				-11.86 (43)			
compound **8** (3S,4R), ring A reactive^b^	-6.51 (33)	15	-12.18 (23)	12	-12.55 (15)	11	-10.68 (18)	9
			-11.71 (28)				-10.48 (38)	
compound **8** (3S,4R), ring B reactive^c^	-6.19 (36)	22	-13.91 (17)	15	-11.61 (26)	8	-12.00 (34)	15
			-13.88 (25)		-11.60 (41)			
Ceftriaxone	-4.99 (30)	16	-14.29 (36)	27	-12.22 (17)	22	-11.75 (4)	28
					-12.00 (20)		-10.65 (12)	

^a^: when the result with the best energy is not associated to the most populated cluster, the energy and the number of poses of the most populated cluster is additionally reported.

^b^: the isolated beta lactam ring has been considered reactive towards the acylation

^c^: the beta lactam ring of the 7-ACA moiety has been considered reactive towards the acylation.

**Table 4:** Results of covalent docking between beta lactam antibiotics and selected PBPs of Gram negative bacteria.

	5FGZ (PBP1b from *E*. *coli*)		4BJP (PBP3 from *E*. *coli*)		2EX8 (PBP4 from *E*. *coli*)		1Z6F (PBP5 from E. coli)		3OCL (PBP3 from *P*. *aeruginosa*)		4K91 (PBP5 from *P*. *aeruginosa*)	
Ligand	Predicted ΔG (kcal/mol) and number of poses in the selected cluster^a^	Total number of clusters	Predicted ΔG (kcal/mol) and number of poses in the selected cluster^a^	Total number of clusters	Predicted ΔG (kcal/mol) and number of poses in the selected cluster^a^	Total number of clusters	Predicted ΔG (kcal/mol) and number of poses in the selected cluster^a^	Total number of clusters	Predicted ΔG (kcal/mol) and number of poses in the selected cluster^a^	Total number of clusters	Predicted ΔG (kcal/mol) and number of poses in the selected cluster^a^	Total number of clusters
compound **8** (3R,4S), ring A reactive^b^	-11.23 (19)	15	-11.17 (49)	12	-11.76 (50)	11	-10.25 (49)	4	-11.62 (36)	10	-10.09 (8)	16
	-10.48 (20)										-9.41 (30)	
compound **8** (3R,4S), ring B reactive^c^	-13.02 (26)	17	-13.36 (49)	13	-12.14 (1)	11	-12.73 (66)	8	-14.30 (51)	13	-12.86 (28)	14
					-11.95 (27)							
compound **8** (3S,4R), ring A reactive^b^	-11.29 (27)	10	-11.83 (41)	16	-10.15 (51)	9	-9.84 (44)	7	-12.29 (25)	9	-10.18 (12)	18
	-11.23 (27)										-9.69 (29)	
compound **8** (3S,4R), ring B reactive^c^	-12.73 (17)	15	-12.48 (25)	17	-13.46 (47)	11	-11.58 (37)	12	-14.16 (64)	9	-13.55 (30)	17
	-12.42 (28)											
Ceftriaxone	-11.87 (4)	30	-12.55 (5)	31	-12.76 (11)	14	-11.33 (29)	17	-13.99 (5)	25	-12.14 (8)	37
	-11.39 (20)		-12.21 (12)		-11.77 (22)				-11.98 (18)		-11.06 (13)	

^a^:when the result with the best energy is not associated to the most populated cluster, the energy and the number of poses of the most populated cluster is additionally reported.

^b^: the additional beta lactam ring has been considered reactive towards the acylation

^c^: the beta lactam ring of the 7-ACA moiety has been considered reactive towards the acylation.

The detailed analysis of the interactions between compound **8** and the binding site of the selected PBPs (S2 Table and S3 Table) shows that H-bonds are prevalent among other kinds of bonds. Indeed, the active site of all PBPs analysed contains several polar residues such as Ser, Thr, Tyr, Asn, but also non-polar residues such as Val and Gly can concur in forming H-bonds via their backbone atoms. The portions of compound **8** more frequently involved in H-bonds are the acethoxymethyl moiety linked in position 3 of the 7-ACA nucleus, the carboxylic acid in position 2 (which often interacts favourably also with positively charged residues in its surroundings), and the carbonyl groups of the azetidinone rings, especially in their open form when they interact with the catalytic Ser residue. Also the carbonyl of the acetylated Ser residue is often an acceptor for H-bonds with active site residues.

The two phenyl rings on the isolated azetidinone, in their turn, are able to form several interactions with hydrophobic residues (Leu, Ala, Pro, Val, Phe, Tyr), but often they can form favorable interactions also with charged residues, involving their delocalized electrons. In particular, in the complexes formed with all the PBPs from Gram negative bacteria, and in 2 out of 3 PBPs from Gram positive bacteria, at least one of these two phenyl rings interact with positively charged residues (mainly Arg, but also Lys). In few cases, also interactions with negatively charged residues (Asp, Glu) are predicted. Therefore, it can be predicted that these two phenyl rings provide favorable interactions within the PBPs active site.

Compared with ceftriaxone (S2 Table and S3 Table), the interactions are similar and involve the same residues, confirming that compound **8** interacts in the same binding site of the known cephalosporin.

Considering the good MIC_50/100_ showed by compound **8** with respect to *S*. *aureus*, we have performed a computational study on PBP2a from a methicillin-resistant *S*. *aureus* (MRSA) strain, in order to predict if compound **8** would be in line of principle active also against this drug-resistant bacterial strain. We selected this protein because it is considered as a key determinant of the broad-spectrum beta lactam resistance in MRSA, because of its reduced affinity for beta lactam antibiotics [34]. We selected the structure of MRSA PBP2a obtained by Bouley and coworkers in complex with a quinazolinone derivative (PDB file: 4CJN) [30] because it was the one which better fit the quality criteria described in Materials and Methods section. The simulation of the interactions between compound **8** and the active site of this protein produced for both antibiotics a negative binding energy. This value is higher in both cases with respect to those predicted for the other PBPs, but the predicted binding energy of compound **8**, on average, was better than the one predicted for ceftriaxone (Table 3). This suggests that this new compound could be more able to bind and inhibit this particular enzyme with respect to the beta lactam antibiotics already available in clinics.

## Discussion

We have synthesized and characterized a new cephalosporin derivative, compound **8**, bearing an additional isolated azetidinone ring in its structure, in analogy with our previous compound derived from penicillin [17]. This compound has been proved to have an antibacterial activity towards Gram positive bacteria, at concentrations comparable or even better than those obtained for the reference antibiotic ceftriaxone, a well known third-generation cephalosporin widely used in clinics. Noteworthy, compound **8** showed better antimicrobial activity compared with ceftriaxone against *Staphylococcus aureus*, one of the main pathogens responsible for a number of infections in hospital settings, with considerable morbidity and mortality. A MIC twice lower than ceftriaxone could make compound **8** potentially more effective in controlling infections from *S*. *aureus*.

Moreover, this compound did not show cytotoxic effects in two selected cell lines routinely used for toxicity evaluation of chemicals [35,36], at concentrations even 10 times higher than those required to have an antimicrobial effect. On the contrary, at the highest concentrations tested, ceftriaxone showed a slight but significant cytotoxic effect on one of the two cell lines.

For all these reasons, compound **8** could be considered as an alternative antibacterial agent that can replace those molecules that are no longer effective as they once were.

The computational analysis indicates that this antibiotic can bind to many different PBPs of both Gram positive and Gram negative bacteria with similar predicted binding energies. This result suggests that the lack of activity of compound **8** towards Gram negative microorganisms (Table 1) may be caused by its inability to cross the external membrane forming the complex cell wall of these bacteria, rather than by its inability to interact with its target. In fact, in Gram negatives, PBPs are mainly located in the periplasmic space of the cell, therefore the antibiotics must overcome a further barrier, whereas in Gram positives, PBPs are external to the cell membrane, near the peptidoglycan, and more exposed to the action of antimicrobial agents [3]. These data suggest that the modifications to render our molecule more active towards Gram negative bacteria should be directed to improve the ability of compound **8** to pass the membrane, rather than to improve its ability to bind its natural targets.

Compound **8** is predicted to bind to PBPs with both azetidinone rings, but the isolated ring seems to have a significantly higher binding energy to the enzyme's active sites in almost all cases, with respect to that predicted for the 7-ACA moiety. Nevertheless, the additional isolated beta lactam moiety can interact favourably with the binding site of all PBPs, as inferred by the positive interactions found in the different complexes obtained by docking, thereby suggesting that the introduction of substituents in the two phenyl rings could modulate the affinity of this moiety towards its target proteins. On the contrary, no significant energetic differences appear in most case to be predicted between the two diastereoisomeric forms of compound **8**, suggesting that the active site of the PBPs might not be selective towards this property.

We have performed a computational analysis also on PBP2a of a MRSA strain in order to predict if compound **8** would be able to face this multi-resistant bacterial strain. The less favourable binding energy predicted both for compound **8** and for ceftriaxone towards this enzyme is in line with its known lower sensitivity with respect to beta lactam antibiotics. It is interesting to note that compound **8** seems to have a better binding energy for this enzyme with respect to ceftriaxone. However, these results should be considered very carefully, considering also the peculiarity of the structure of PBP2a selected for these simulations. In fact, this structure contains two active sites: the common DD-transpeptidase active site, with the catalytic residue Ser 403, and an allosteric site to which the quinazolinone is (surprisingly) bound. The two chains composing this structure show a difference in the conformations of two loops near the transpeptidase active sites, attributed to the effect of the interaction of the quinazolinone derivative with the allosteric site in the structure, with a “closed” conformation in the chain A, and an “open” conformation in chain B [30]. We made attempts in order to repeat the docking using other structures of MRSA PBP2a available [34], but all of them have missing residues in the active site, and we failed to reproduce the correct binding of their co-crystallized ligands. Therefore, we had to conclude that these last proteins are unreliable to study the interactions with the current covalent docking procedure.

In conclusion, compound **8** is a promising molecule to develop a new class of safe and active antibiotics, with a predicted good binding energy for PBPs not only for Gram positive, but also for Gram negative microorganisms. For the future, we are planning to synthesize and test a series of derivatives of compound **8** with the scope of obtaining molecules with an increased potency for both azetidinone rings and a wider spectrum of action, possibly covering also multidrug resistant bacterial strains.

## Materials and methods

### Chemistry

All reagents and anhydrous solvents were obtained from commercial sources and used without further purification. All reactions requiring anhydrous conditions were performed under N_2_ atmosphere and all glassware were flame dried. Thin-layer chromatography was performed on Macherey-Nagel pre-coated aluminum sheets (0.20 mm, silica gel 60 with fluorescent indicator UV254) in appropriate solvent. Column chromatography was conducted using silica gel (70–230 mesh, Merck). ^1^H and ^13^C-NMR spectra were recorded on Bruker DRX 400, 600 spectrometers (600 MHz, 400 MHz for ^1^H; 151 MHz, 100 MHz for ^13^C). *J* values are given in Hz. Chemical shifts were reported as δ value, and referenced to the solvent peak: CDCl_3_ (77.0 ppm). For D_2_O, ^13^C-NMR spectra were referenced to the signal for the carbonyl group of acetone (one drop, added as an internal standard), which was set to 215.94 ppm. ESI-(+)-MS measurements were acquired on a Waters 4 micro quadrupole mass spectrometer equipped with electrospray ion source. Elemental analysis was performed on the FlashEA 1112 Series with Thermal Conductivity Detector. IR spectra were recorded on an FT-IR instrument Bruker Vector 22. Melting points were performed on DSC 2920 TA INSTRUMENTS. Compounds **3**, **5**, **6** and **7** were prepared following the previously described methodology and their spectral data matched with those reported in literature [17].

#### Synthesis of sodium (6R, 7R)-3-(acetoxymethyl)-8-oxo-7-(2-(2-oxo-1,4-diphenylazetidin-3-yl)-acetamido-5-thia-1-azabicyclo[4.2.0]oct-2-ene-2-carboxylate (compound 8)

To a solution of (+/-)-*trans*-**7** (80.4 mg, 0.30 mmol) in anhydrous dichloromethane (3.8 mL) at room temperature, was added DCC (68 mg, 0.33 mmol, 1.1 equiv). The mixture was stirred for 30 min. and then filtered on Celite pad in a solution of 7-ACA (90.0 mg, 0.33 mmol, 1.1 equiv) and Et_3_N (33.4 mg, 0.33 mmol, 1.1 equiv) in anhydrous dichloromethane (1.7 mL). The reaction mixture was stirred at room temperature overnight. The mixture was filtered and the filtrate was washed with distilled water. The organic phase was dried over Na_2_SO_4_, filtered and concentrated under reduced pressure. The resulting crude was solubilized in dichloromethane and crystallized from hexane in an ice bath. The solid was dissolved in dichloromethane (1.0 mL), washed with a 0.1 M HCl solution (0.5 mL), next with distilled water until the washes were about pH 5, and finally was dried over Na_2_SO_4_. The solvent was removed under vacuo, and the residue was dissolved in acetone (0.5 mL) with an aqueos solution (1.8 mL) of NaHCO_3_ (2 equiv). After 15 min the mixture was diluted with ether. The aqueous phase was separated and freeze-dried affording in high purity the compound **8** as a sodium salt (55% yield).

Solid amber as inseparable mixture of diastereomers, dr = 7:3 (see Supporting Information). m.p. 105–108°C. IR (KBr pellet, cm^-1^): ν 3400, 2924, 1751, 1662, 1592, 1389, 1503. ^1^H-NMR (D_2_O, 600 MHz) δ: 7.38–7.04 (20H, m), 5.62 (2H, d, *J* = 3.5 Hz), 5.09–5.03 (4H, m), 5.00 (1H, bs), 4.88–4.84 (2H, m), 4.73–4.69 (2H, d), 3.57–3.51 (2H, m), 3.49–3.46 (1H, m, minor diastereoisomer), 3.45–3.41 (1H, m, major diastereoisomer), 3.29–3.23 (2H, m), 3.00–2.84 (4H, m), 2.09 (3H, s, minor diastereoisomer), 2.08 (3H, s, major diastereoisomer) (S1 Fig). ^13^C-NMR (151 MHz, D_2_O): δ, 174.0, 173.0, 172.96, 168.6, 168.4, 164.7, 136.7, 136.4, 131.5, 129.3, 129.1, 128.8, 126.5, 124.9, 117.8, 116.4, 116.3, 64.2, 64.1, 60.6, 60.3, 59.2, 59.1, 57.2, 55.2, 55.0, 33.0, 25.4, 20.3 (S3 Fig). ESI (+)-MS: m/z 557 [M+Na]^+^. Anal. calcd. for C_27_H_24_N_3_O_7_SNa: C: 58.16, H: 4.34, N: 7.54; found: C: 58.14, H: 4.35, N: 7.51. Purity of compound **8**. Final compound **8** purity was determined by elemental analysis on a FlashEA 1112 Series with Thermal Conductivity Detector, for C, H, and N. The final purity of compound **8** was found to be >95% when analyzed.

### Microbiological assays and bacterial strains

The MIC and the MLD of antibiotics were estimated using the densities (5 x 10^5^ CFU/mL) and protocols recommended by the Clinical and Laboratory Standards Institute (CLSI), formerly the National Committee for Clinical Laboratory Standards (NCCLS) [17, 22]. The effects of antibiotics concentrations on the microbial growth were evaluated by turbidity, by measuring optical density at 600 nm (OD_600_). MIC_100_ was defined as the minimum antibiotic concentration that does not change the sample turbidity compared to time 0, while the MIC_50_ as the minimal concentration that reduced OD_600_ of 50% compared to that of inoculated sample without antibiotic. MLD was estimated by CFU/mL determination. Briefly, for each examined antibiotic, different dilutions of each bacterial inoculum growth for 24 h in the presence of antibiotic concentrations ≥ MIC_100_ were spread on plate count agar (PCA) and CFU were counted after incubating 24 h at 37°C. The MLD was the minimum antibiotic concentration at which the number of CFU/mL resulted equal to 0. The in vitro antibacterial activity of the new compound was compared to that of the ceftriaxone (Sigma-Aldrich, Milan, Italy), a reference cephalosporin agent active towards a broad range of Gram positive and Gram negative bacteria, in clinical use for the treatment of a variety of infections such as meningitis, gonorrhea and community-acquired pneumonia.

Bacterial strains used for in vitro antibiotic activity determination included pathogenic and not pathogenic isolated, both Gram negative *Escherichia coli*, *Salmonella*, and Gram positive *Staphylococcus aureus* and *Bacillus* sp. *E*. *coli* (strain JM109) was purchased from Promega (http://www.promega.com/products, accessed December 4,2015). *Salmonella enterica* subsp. *Enterica* serovar *Typhimurium*, strain LT2 ATCC 700720 was purchased from Leibniz Institute DSMZ-German Collection of Microorganisms and Cell Culture (http://www.dsmz.de, accessed December 4, 2015). The remaining strains were derived from the collection deposited in the microbiology laboratory directed by dr. G. Vigliotta.

### Cytotoxicity assays on human cells

Cytotoxicity of compound **8** was monitored by the MTT (Sigma-Aldrich) colorimetric test [37] performed on two different human cell lines, i.e. MRC5, a hembryonic lung cell line, and HepG2, a hepatoma cell line, both obtained from Interlab Cell Line Collection (IST, Istituto Nazionale per la Ricerca sul Cancro, Genoa, Italy). Cells were cultured at 37°C in a 5% CO_2_, 95% air-humidified atmosphere in Dulbecco’s modified Eagle’s medium ‎(MRC5) or Essential’s modified Eagle’s medium (HepG2) supplemented with 10% foetal bovine serum, 0.2 mM L-glutamine, 50 units/mL penicillin and 50 μg/mL streptomycin (Sigma-Aldrich). The medium for HepG2 cells was also supplemented with 1% non-essential amino acids.

Compound **8** stock solution (10 mg/ml) was prepared in 80% DMSO (Sigma-Aldrich). Ceftriaxone (Sigma-Aldrich) stock solution (25 mg/mL) was prepared in deionized filtered H_2_O. For treatments, cells were seeded at the density of 6.5x10^4^/cm^2^ in 96-well plates and cultured for 24 h. Then different amounts of compound **8**, or ceftriaxone, were added to the wells and cells were incubated for further 24 h. Finally, the MTT solution (2.5 mg/mL in sterile phosphate buffer saline) was added to each well (final concentration 0.125 mg/mL) and incubated for 1 h at 37°C to allow formazan crystals deposition. Crystals were dissolved in DMSO and absorbances were measured at 595 nm. Cell viability was calculated by comparing absorbances registered in the wells added with compound **8** (or ceftriaxone) and absorbances registered in the wells added with corresponding amounts of vehicle only. Data were plotted as percent of mean values ± SD. The Student’s test was performed to identify statistically significant differences (at p < 0.05). As positive control of the assay, 0.05% H_2_O_2_ was used for 15 min.

### Computational section

The structures of the PBPs used for the present work were downloaded from the Protein Data Bank [38] using as a reference structure the biological assembly as determined by the authors. We have selected representative proteins for each class of the PBPs belonging to both Gram positive and Gram negative bacteria; when available, we selected the PBPs belonging to the same microorganisms used to determine the MIC [24–33]. Since many different structures of the same protein in complex with different ligands and/or obtained in different experimental conditions were available, the selection of the representative proteins was made on the basis of many quality criteria, including: the completeness of the structure (absence of missing residues and atoms at least in the active site), the absence of mutations in the active site, resolution (better than 2.85 Å), acceptable R-value and R-free, analysis of the distribution of residues into the Ramachandran plot and of the RMS deviation of the bond lengths and angles from ideal values, low average and local B-values. Additionally, two web-servers were used to evaluate the quality of the structures: ProSA-Web [39], and Q-MEAN [40]. The final list of selected structures is reported in Table 2. In some selected structures, there were some residues with multiple conformation identified (occupancy <1). In these cases, each conformation was analyzed separately.

The structures of the known antibiotics used as a reference were either extracted from the selected complexes downloaded from the PDB, or (in the case of ceftriaxone) downloaded from PubChem [41] in 3D.sdf format, then converted into the.pdb format by using Chimera [42]. Chimera was also used to modify the ligand, to add hydrogens and charges, to prepare it for the covalent docking and, if necessary, to optimize the modified structure for the following steps. The structure of compound **8** was designed and saved in 3D.pdb format by using ChemDraw and Chem3D Pro 12.0 (Perkin Elmer), and then modified in the same way as the other beta lactam antibiotics, to render it suitable for the covalent docking procedure. Since the chemical synthesis of compound **8** produces a diastereoisomeric mixture of the compound, we designed and analysed separately the two diastereoisomers (3R, 4S) and (3S, 4R). Moreover, since potentially both beta lactam rings present in the molecule can react and acylate the catalytic Ser residue, for each diastereoisomer we created and tested the two possible options. Therefore, for each protein, four different results of the docking with compound **8** were obtained. Finally, the 7-ACA moiety was considered both in its undissociated and dissociated form. The results obtained in both cases were generally not significantly different neither in terms of predicted binding energy nor in terms of interactions between compound **8** and the active site of the selected PBPs. In this article we therefore report the results of the undissociated form only.

To perform covalent docking, we adopted the flexible side chain approach recently developed by Bianco and coworkers [23] and implemented in the popular program AutoDock v. 4.2 [43]. In this method, a ligand is modified by opening the beta lactam ring and adding the C = O group at the site of alkylation. These two atoms are then overlapped with the matching atoms in the receptor structure, to form the covalent bond with the Ser residue in the protein's active site before running the docking. The complex is therefore treated as a fully flexible residue in the interior of the protein, and its position and conformation is optimized similarly to a traditional flexible docking procedure. This method reached 75% success rate in reproducing the experimental coordinates in the test set used by the authors [23]. To check for the reliability of this approach and of the parameters used on our dataset, and to give an estimate of the resulting binding energies in the case of known antibiotics, three selected complexes formed by a PBP and a known beta lactam antibiotic (see Table 2) were used to perform a self-docking prediction before running the simulations on compound **8**. The solutions obtained were then compared with the original crystallographic structure (see Results).

For the covalent docking procedure, polar hydrogens were added to the protein and ligands, and charges were assigned according to Gasteiger [44]. Water molecules and the ligands present in the crystallographic structures were removed before performing docking. A grid centered on the catalytic Ser residue and including the residues of the active site reported for each selected structure was set up. The maximum dimension of the grid was 70x70x70 points with a grid spacing of 0.375 Å. For each compound, 100 docking runs were performed using the AutoDock Lamarckian genetic algorithm, leavingthe others parameters as default. An RMSD value of 2.0 Å was taken for clustering the resulting docking poses. The conformations corresponding to the best energetic and the most populated cluster of poses obtained from covalent docking were selected, saved in.pdb format and investigated for the interactions between the enzyme and the beta lactam antibiotic, by using the tools available in the Discovery Studio software (DassaultSystèmes BIOVIA, Discovery Studio Modeling Environment, Release 4.5, San Diego: DassaultSystèmes, 2015).

## Supporting information

S1 Table Results of the self-docking analysis performed on selected complexes between PBPs and bετα lactam antibiotics. (DOCX)

S2 Table Details of the interactions between compound 8, ceftriaxone and each PBP from Gram positive bacteria. In this table are reported for each protein the interactions of the representative pose(s) reported in Table 3. See the legend below each scheme to understand the different interactions.
(DOCX)

S3 Table Details of the interactions between compound 8, ceftriaxone and each PBP from Gram negative bacteria. In this table are reported for each protein the interactions of the representative pose(s) reported in Table 4. See the legend below the scheme to understand the different interactions.
(DOCX)

S1 Fig ^1^H-NMR spectrum of compound 8. (DOCX)

S2 Fig 2D-COSY spectrum of compound 8. (DOCX)

S3 Fig ^13^C-NMR spectrum of compound 8. (DOCX)

S4 Fig 2D HMQC spectrum of compound 8. (DOCX)

S5 Fig **Superposition of the best result obtained with the self-docking procedure on selected complexes, and the crystallographic complex, for: A) 2EX8; b) 3OCL; c) 3VSL.** The ligand is represented in sticks. Red: crystallographic conformation. Blue: best pose obtained by the covalent docking strategy. The picture has been prepared using PyMol Molecular Graphics System, v. 1.7.4. (Schrödinger, LLC).
(DOCX)
