# Supplementary material for: Synthesis and biological evaluation of the progenitor of a new class of cephalosporin analogues, with a particular focus on structure-based computational analysis
Source: PLoS One. 2017 Jul 27;12(7):e0181563. doi: 10.1371/journal.pone.0181563 (PMC5531512; doi:10.1371/journal.pone.0181563)
Supplement: S1 Table — (DOCX) [file pone.0181563.s001.docx]

**S1 Table: results of the self-docking analysis performed on selected complexes between PBPs and β-lactam antibiotics.**

| **PDB code** | **Predicted ΔG (kcal/mol)** | **Number of poses in the selected representative cluster** | **Total number of different clusters** |
| --- | --- | --- | --- |
| 2EX8  (*E. coli* PBP4 + penicillin G) | -9.20 | 99 | 2 |
| 3OCL  (*P. aeruginosa* PBP3 + carbenicillin) | -10.02 | 63 | 7 |
| 3VSL  (*S. aureus* PBP3 + cefotaxime) | -12.54  -12.32 | 58  31 | 5 |
