# Supplementary material for: Synthesis and biological evaluation of the progenitor of a new class of cephalosporin analogues, with a particular focus on structure-based computational analysis
Source: PLoS One. 2017 Jul 27;12(7):e0181563. doi: 10.1371/journal.pone.0181563 (PMC5531512; doi:10.1371/journal.pone.0181563)
Supplement: S2 Table — In this table are reported for each protein the interactions of the representative pose(s) reported in Table 3. See the legend below each scheme to understand the different interactions. (DOCX) [file pone.0181563.s002.docx]

**S2 Table: details of the interactions between compound 8, ceftriaxone and each PBP from Gram positive bacteria.**

In this table are reported for each protein the interactions of the representative pose(s) reported in Table 3. See the legend below each scheme to understand the different interactions.

**3VSL (PBP3 from S. aureus)**

| **Ligand** | **ΔG (kcal/mol)** | **Scheme** |
| --- | --- | --- |
| compound 8 (3R4S), ring A reactive | -12.77 | 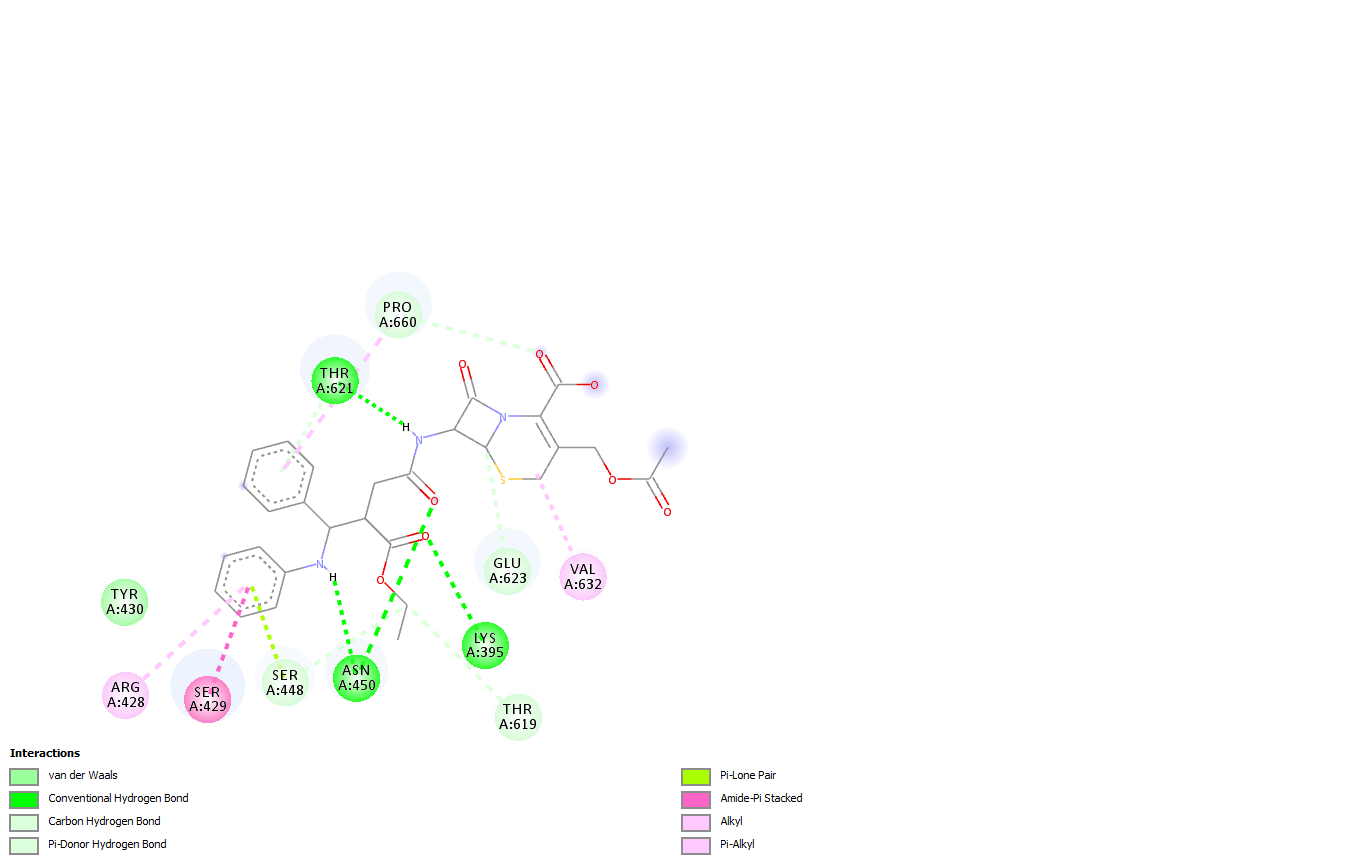 |
|  | -11.82 | 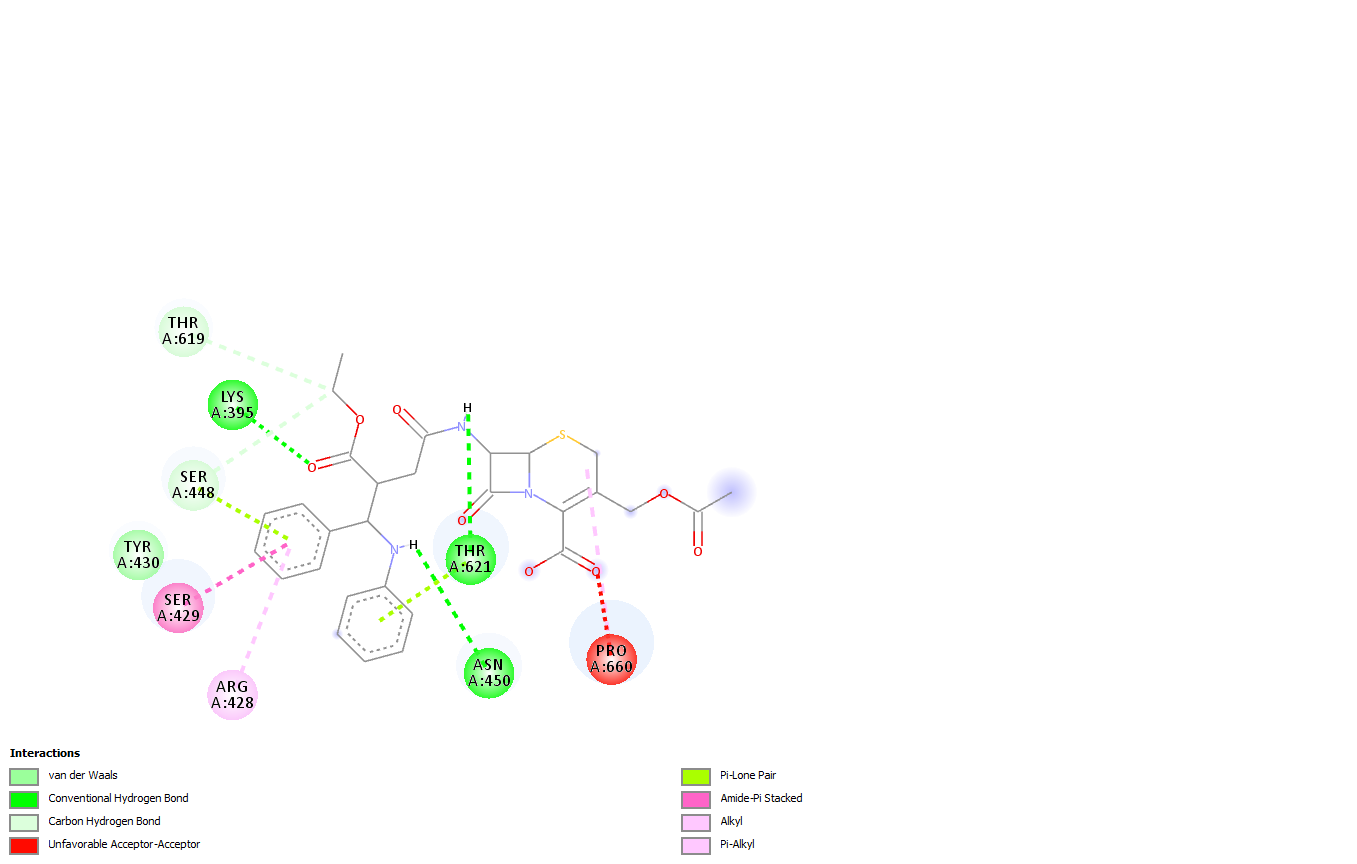 |
| compound 8 (3R4S), ring B reactive | -13.88 | 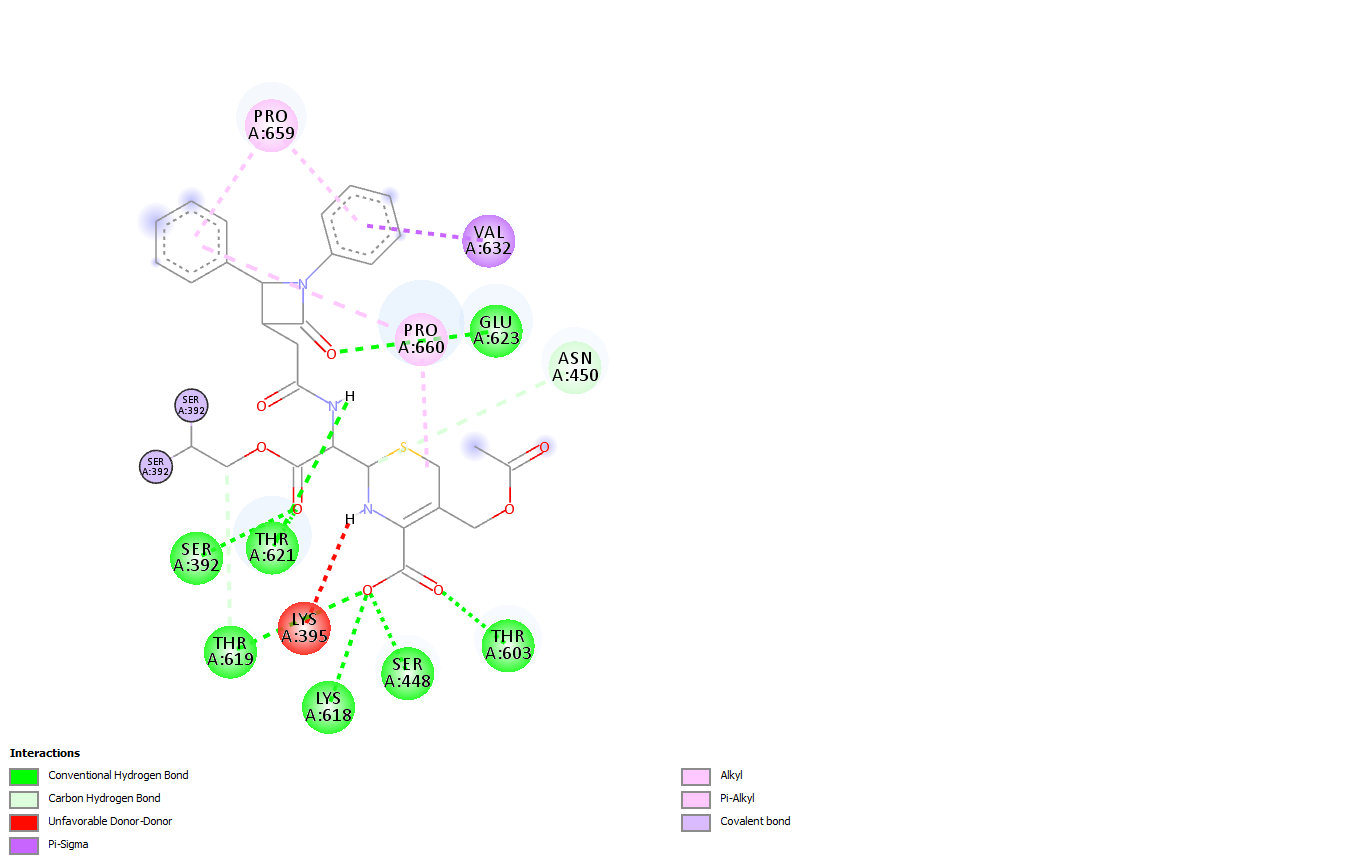 |
| compound 8 (3S4R), ring A reactive | -12.18 | 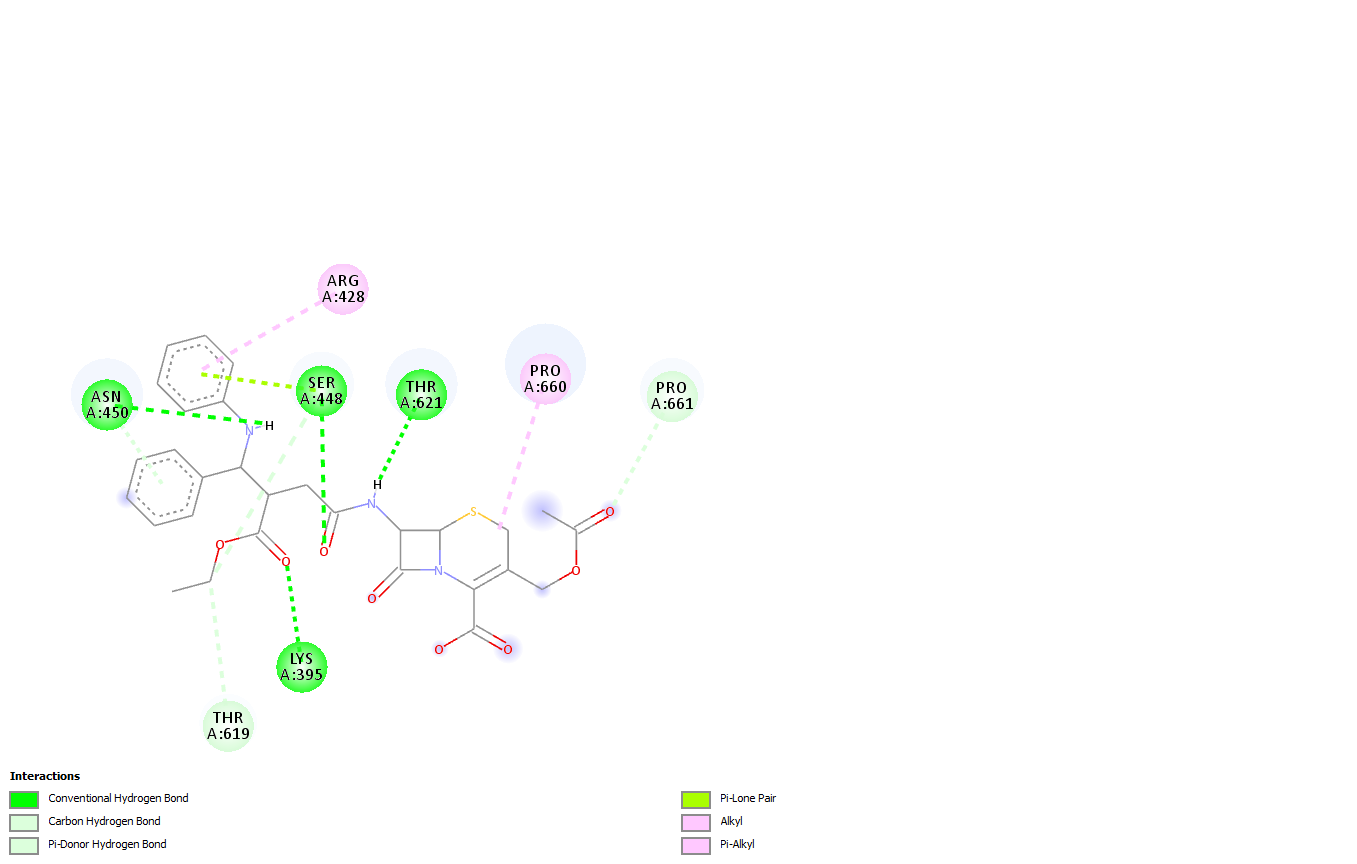 |
|  | -11.71 | 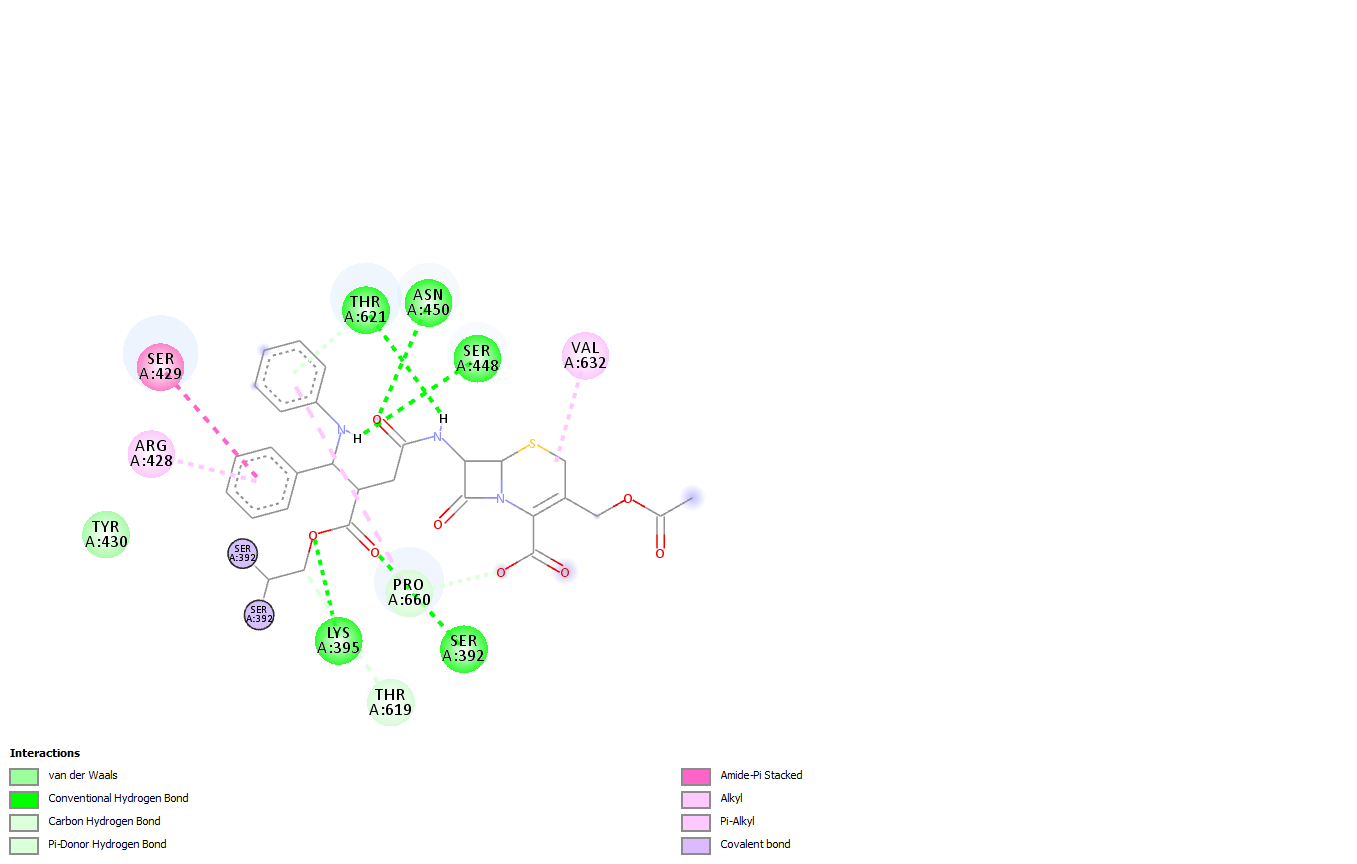 |
| compound 8 (3S4R), ring B reactive | -13.91 | 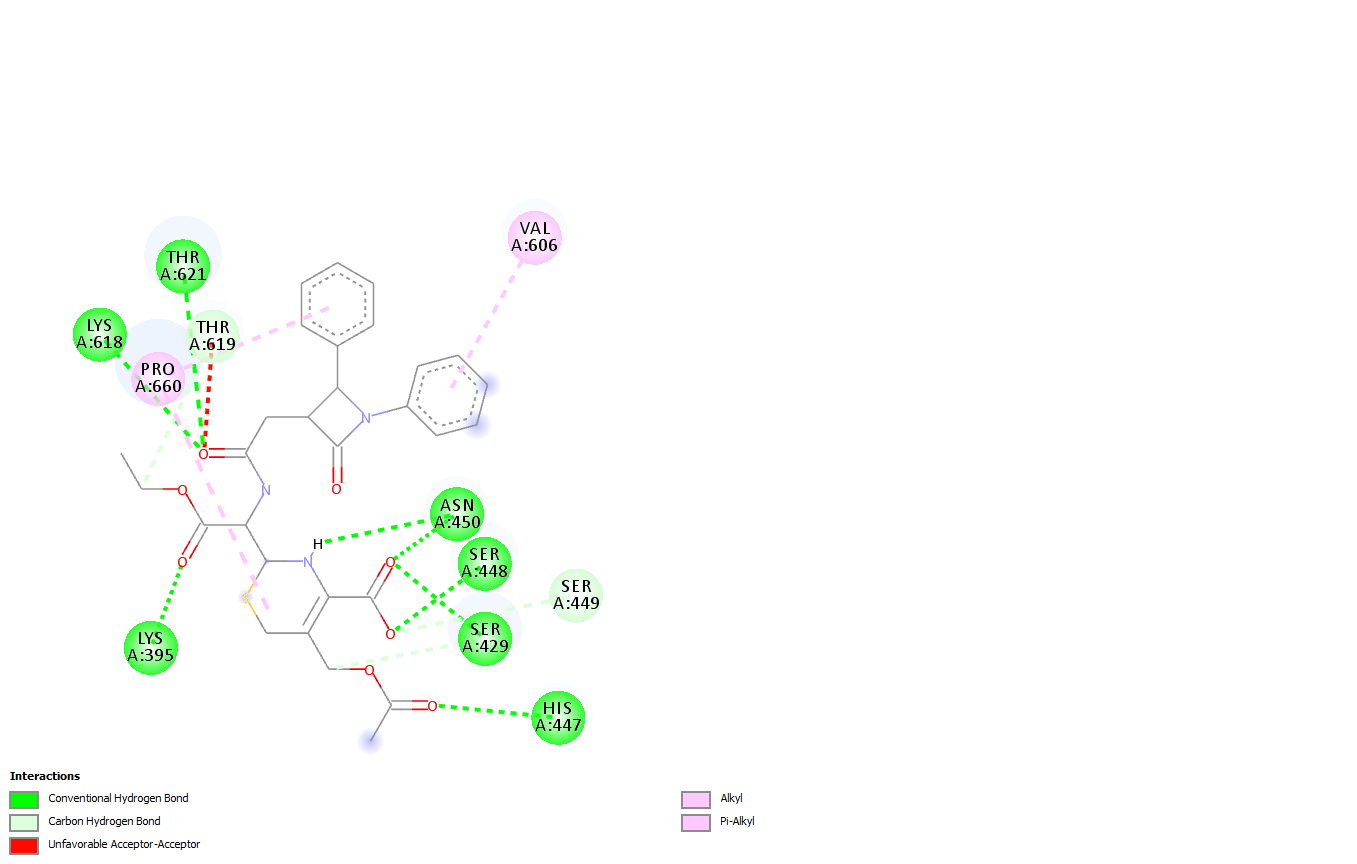 |
|  | -13.88 | 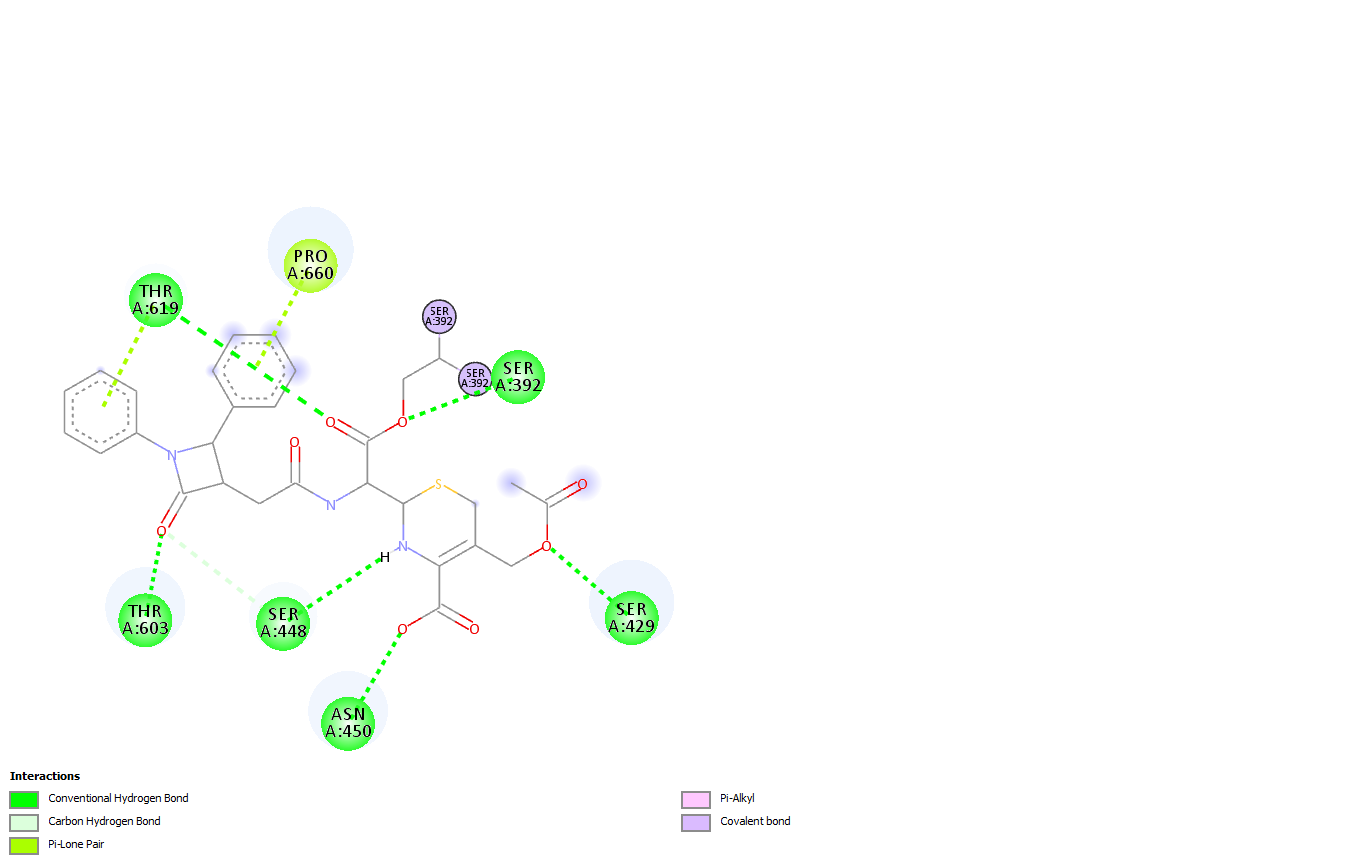 |
| Ceftriaxone | -14.29 | 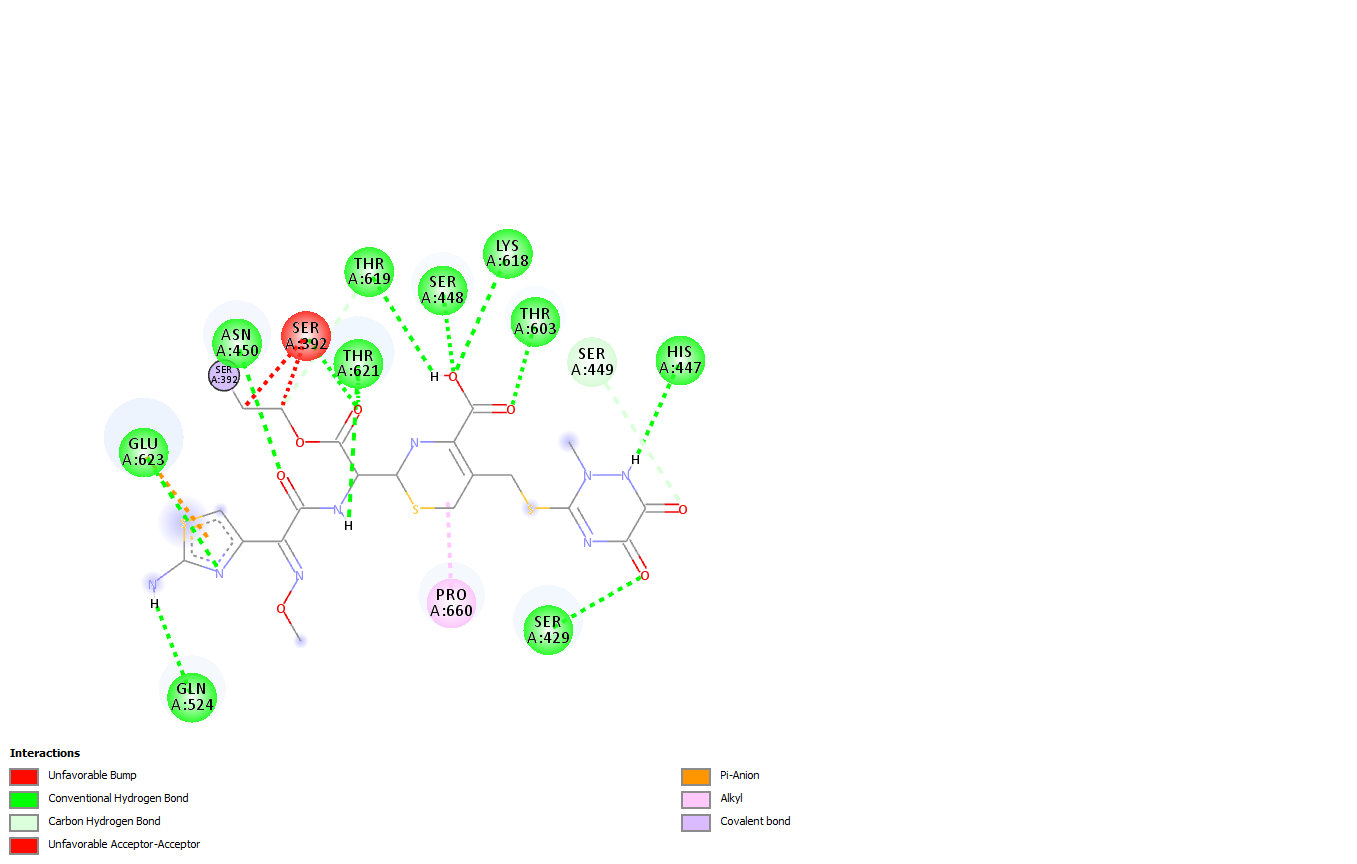 |

**1TVF (PBP4 from *S.* *aureus*) (Suppl. Table 2 – cont.)**

| **Ligand** | **ΔG (kcal/mol)** | **Scheme** |
| --- | --- | --- |
| compound 8 (3R4S), ring A reactive | -12.71 | 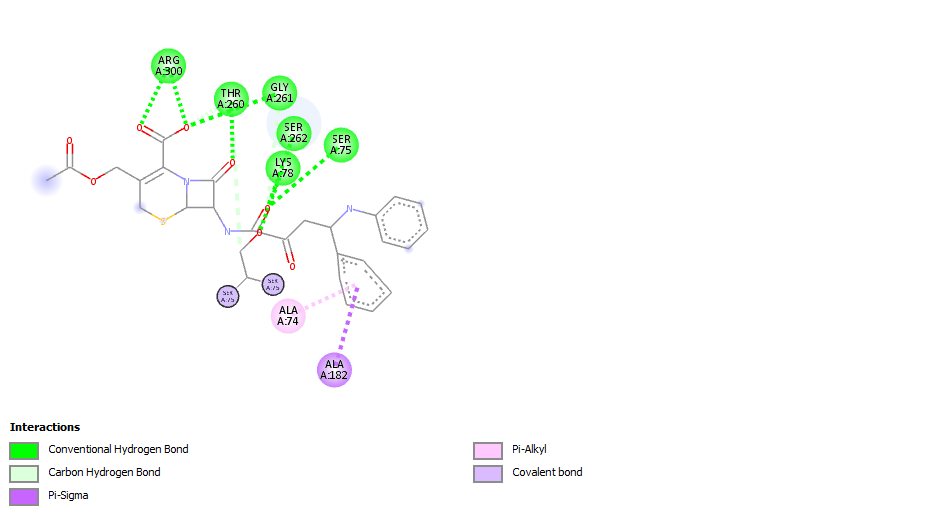 |
| compound 8 (3R4S), ring B reactive | -11.94 | 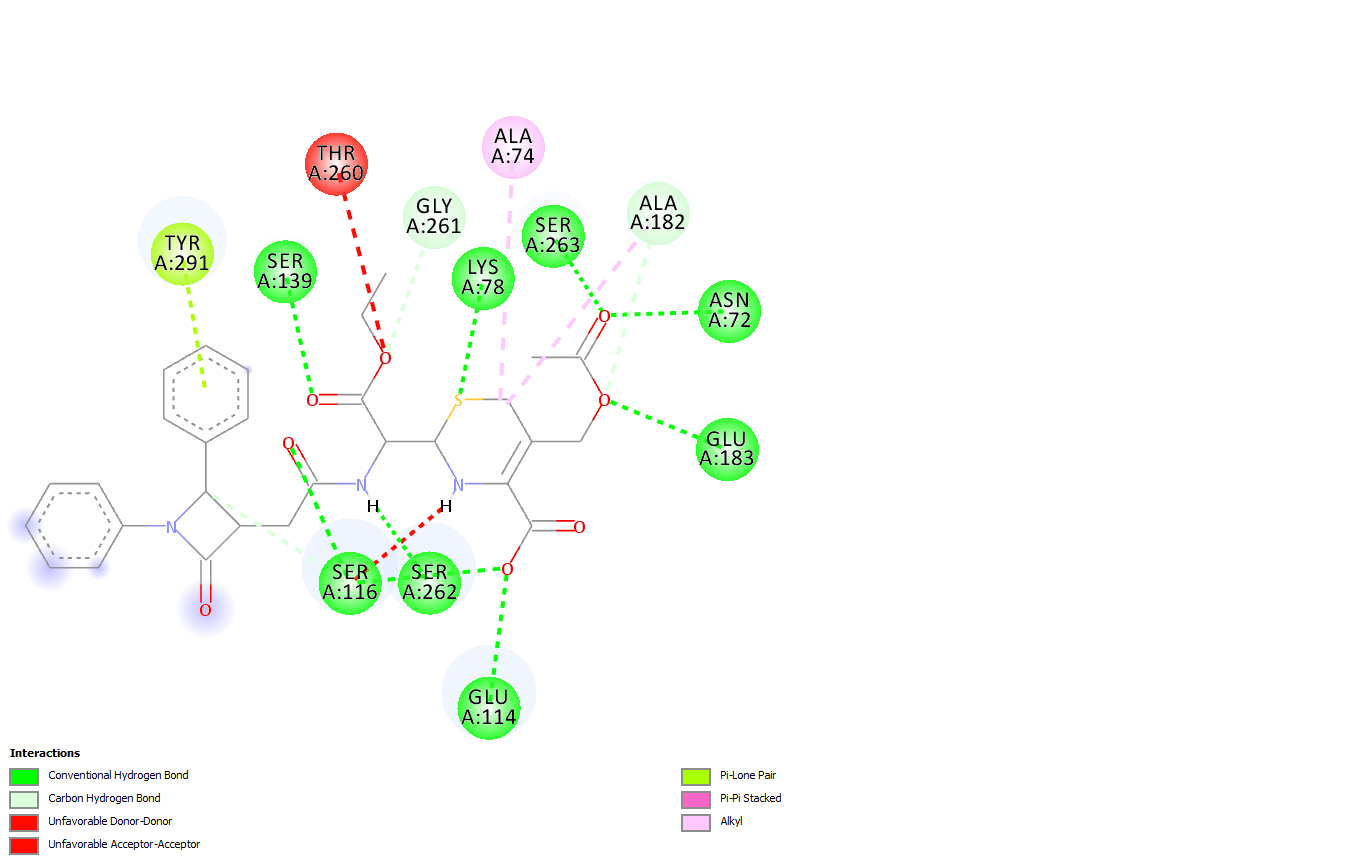 |
|  | -11.86 | 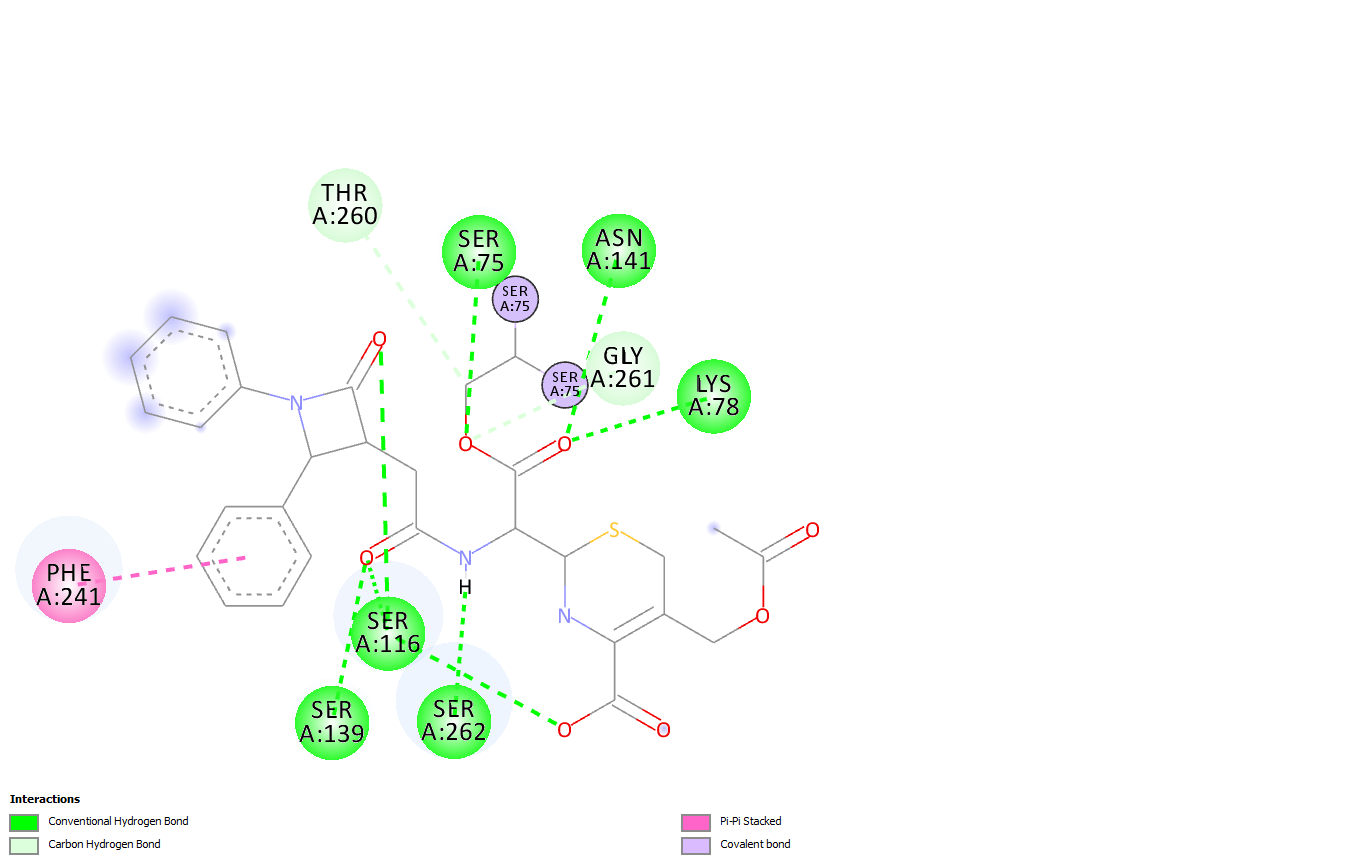 |
| compound 8 (3S4R), ring A reactive | -12.55 | 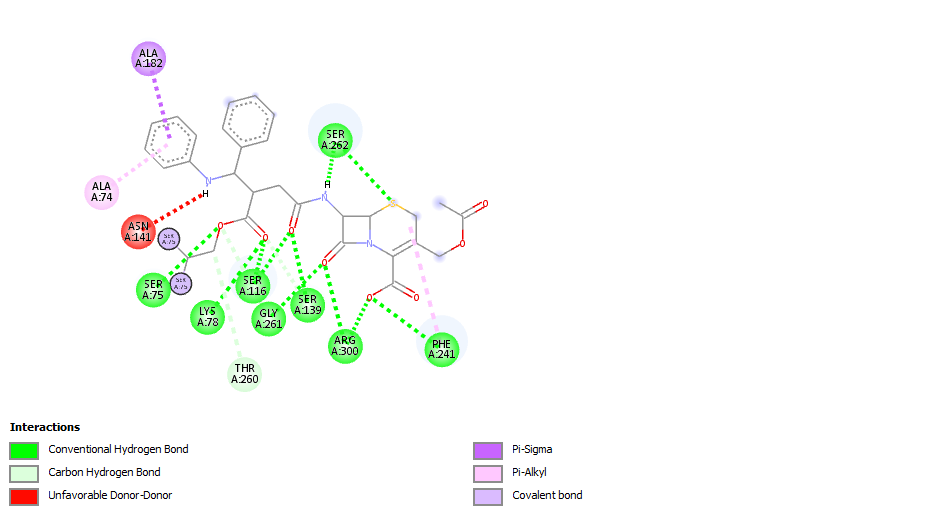 |
| 3S4R_B  compound 8 (3S4R), ring B reactive | -11.61 | 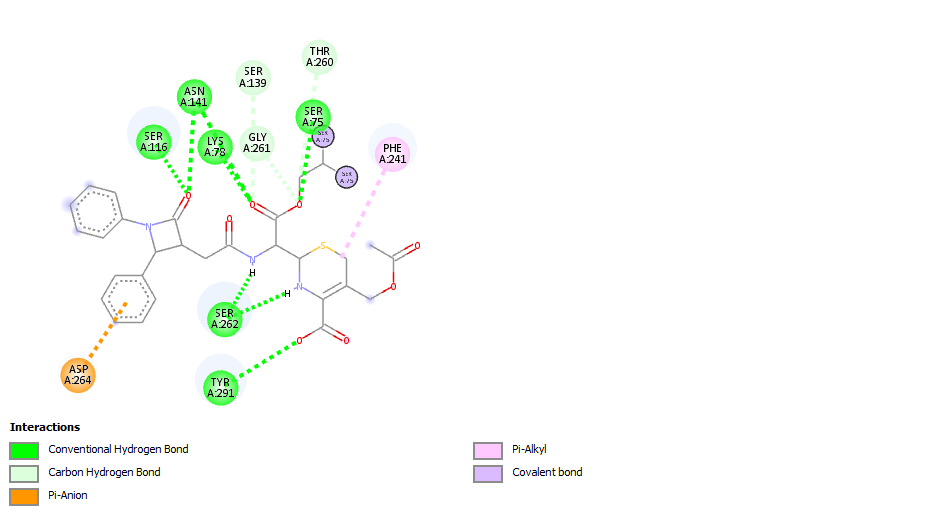 |
|  | -11.60 | 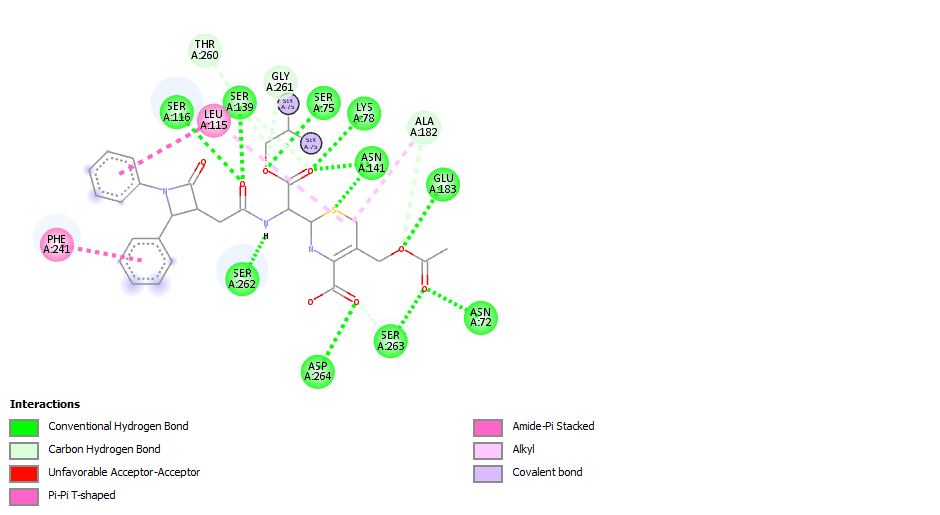 |
| Ceftriaxone | -12.22 | 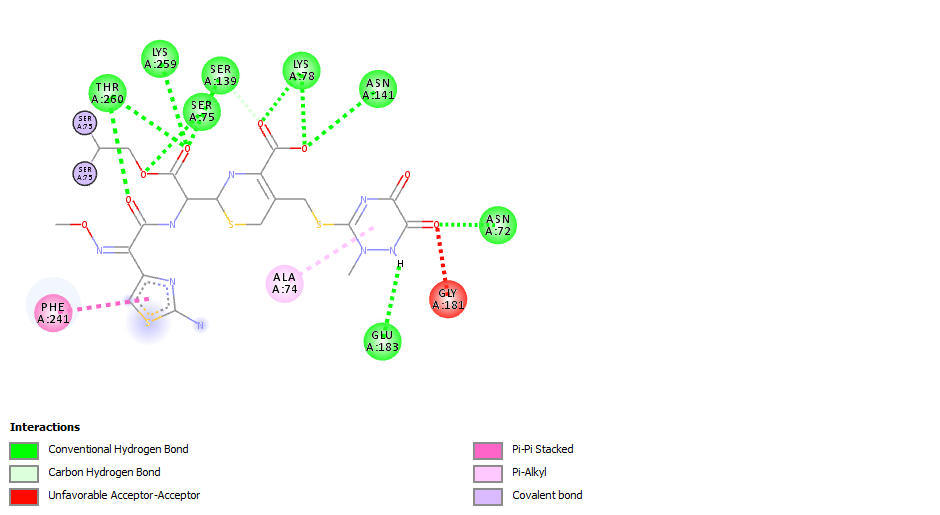 |
|  | -12.00 | 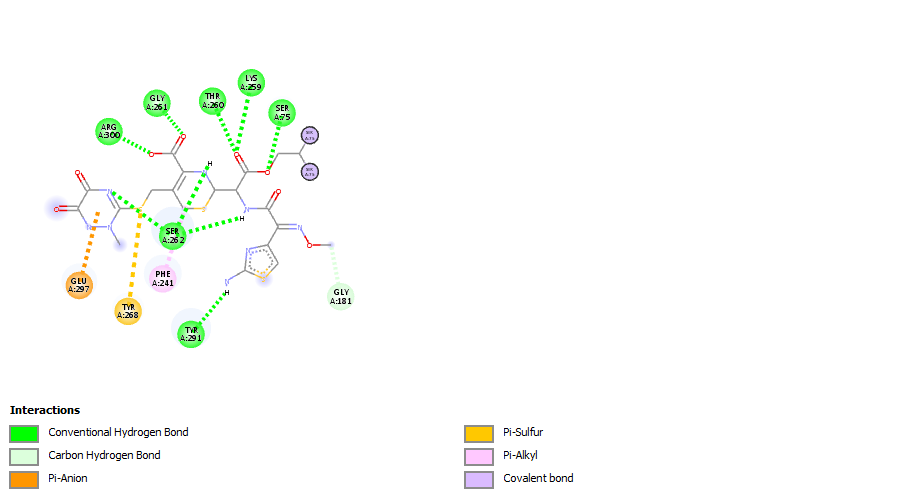 |

**2J9P (PBP4a from *B. subtilis*) (Suppl. Table 2 – cont.)**

| **Ligand** | **ΔG (kcal/mol)** | **Scheme** |
| --- | --- | --- |
| compound 8 (3R4S), ring A reactive | -10.27 | 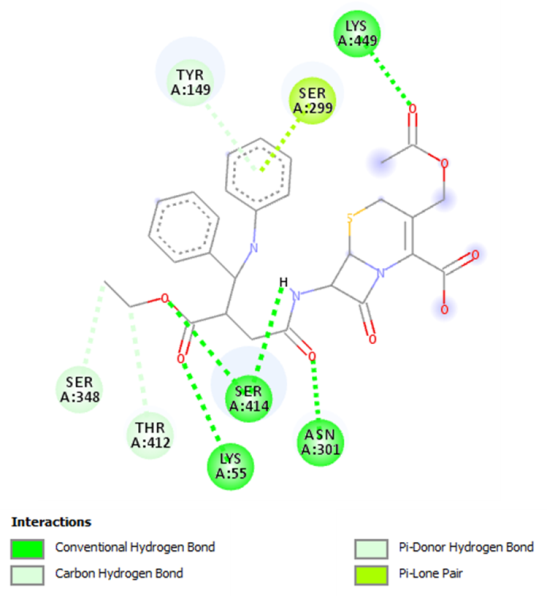 |
|  | -9.48 | 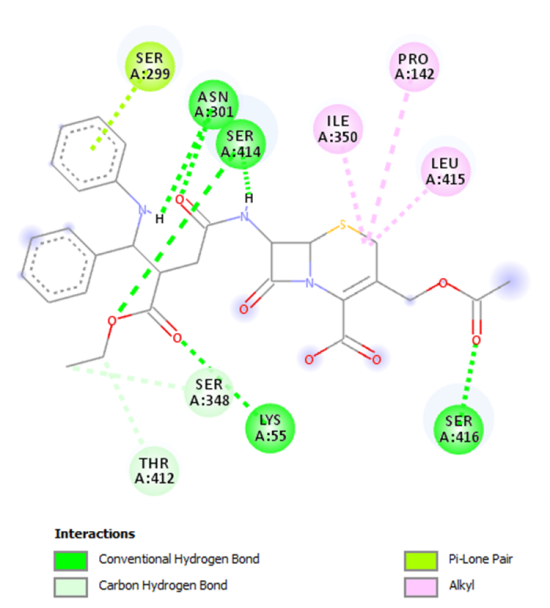 |
| compound 8 (3R4S), ring B reactive | -12.05 | 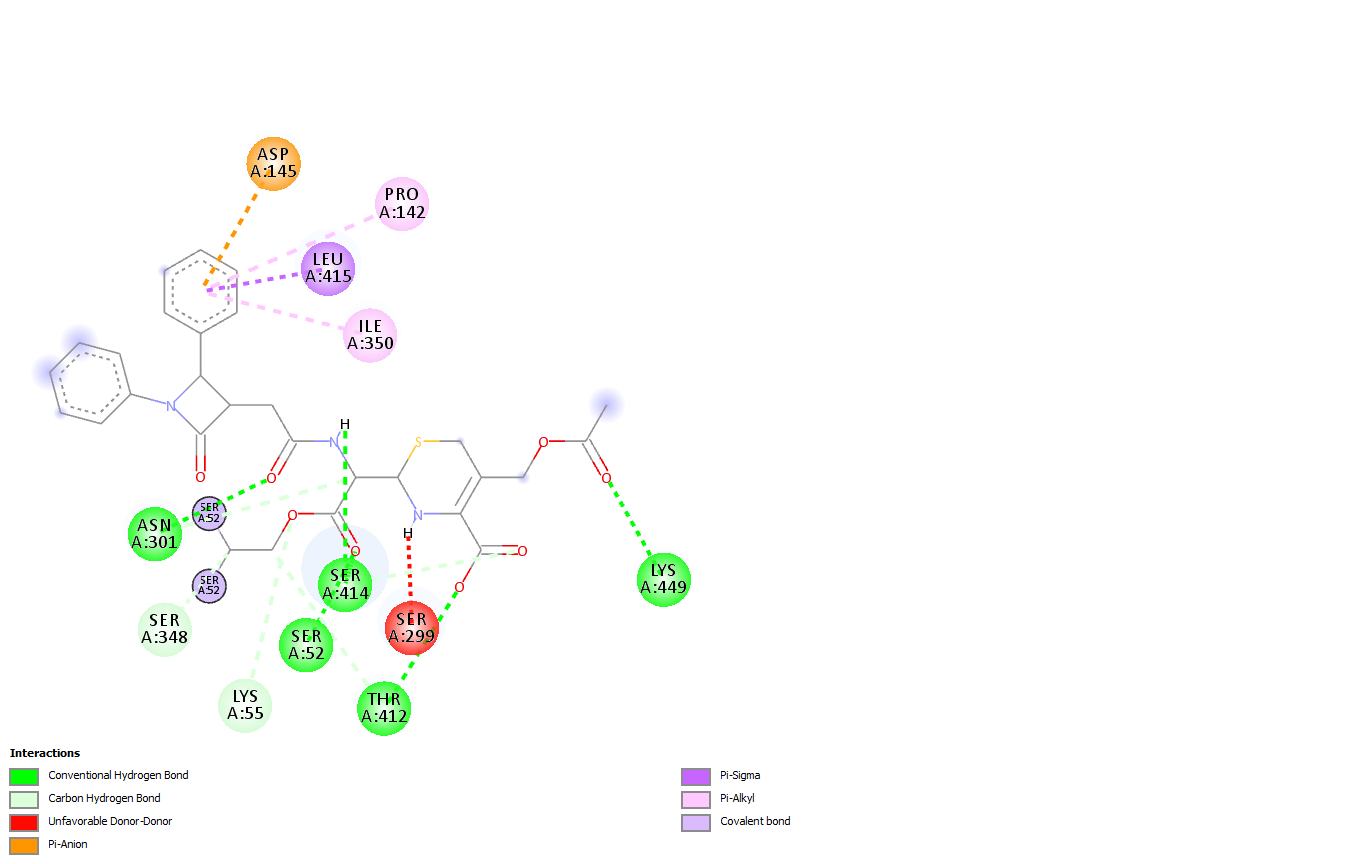 |
| compound 8 (3S4R), ring A reactive | -10.68 | 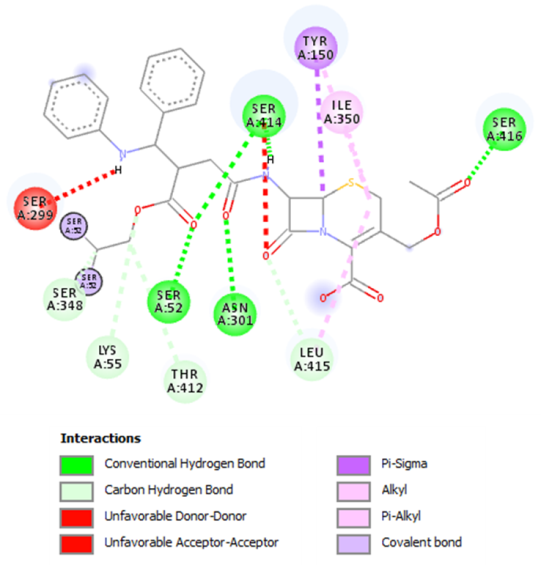 |
|  | -10.48 | 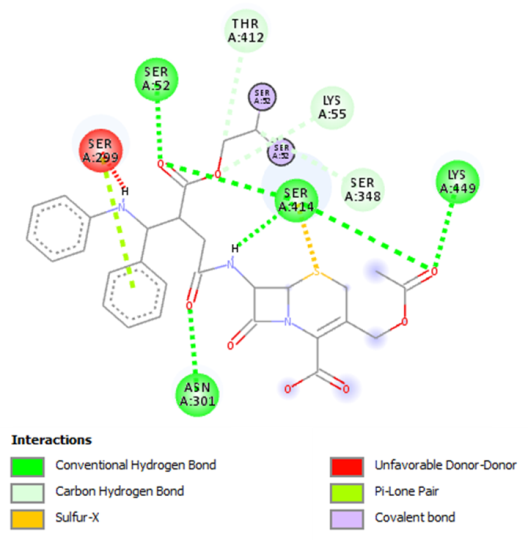 |
| compound 8 (3S4R), ring B reactive | -12.00 | 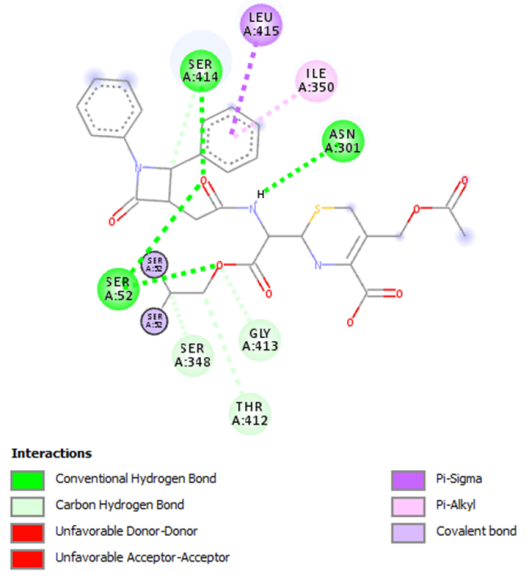 |
| Ceftriaxone | -11.75 | 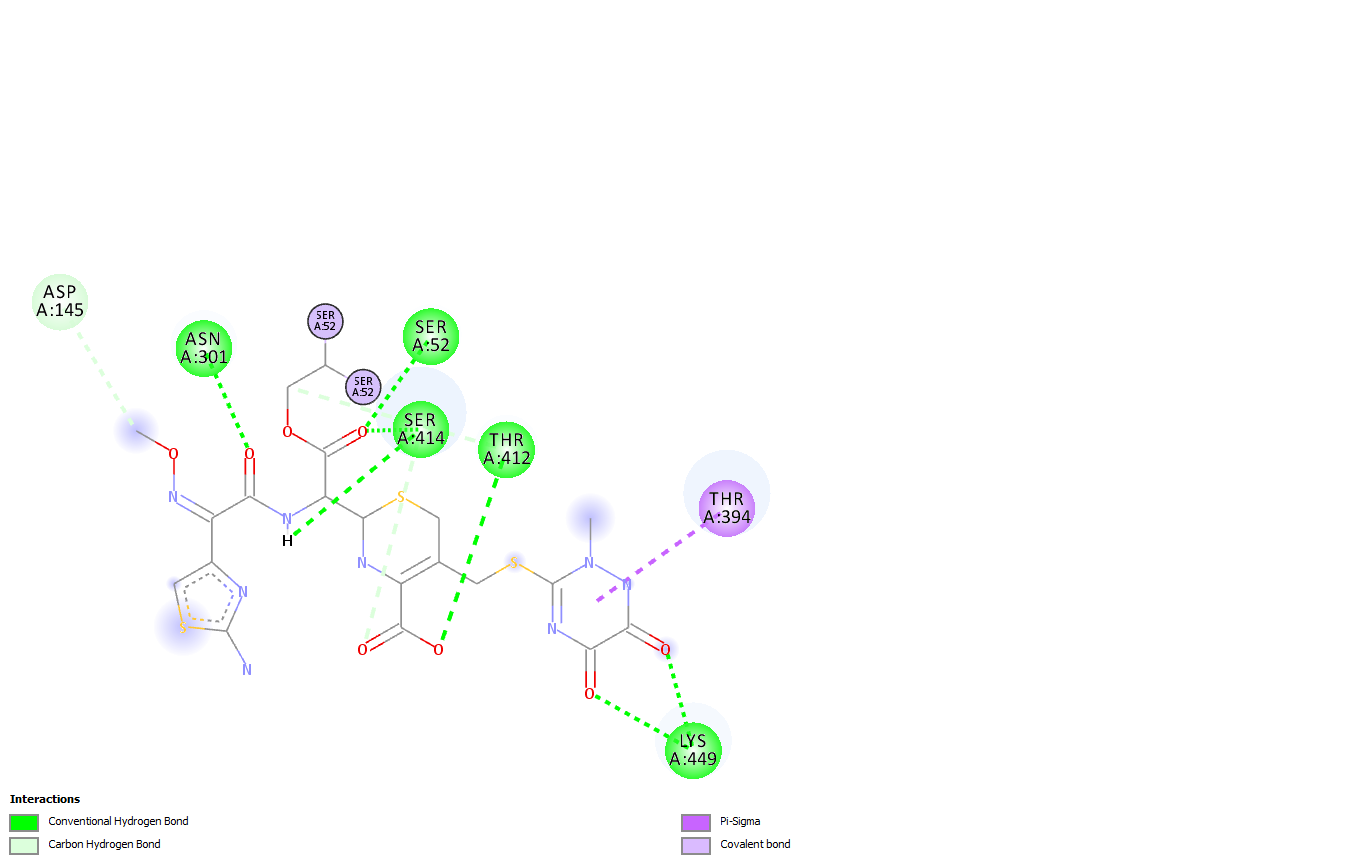 |
|  | -10.65 | 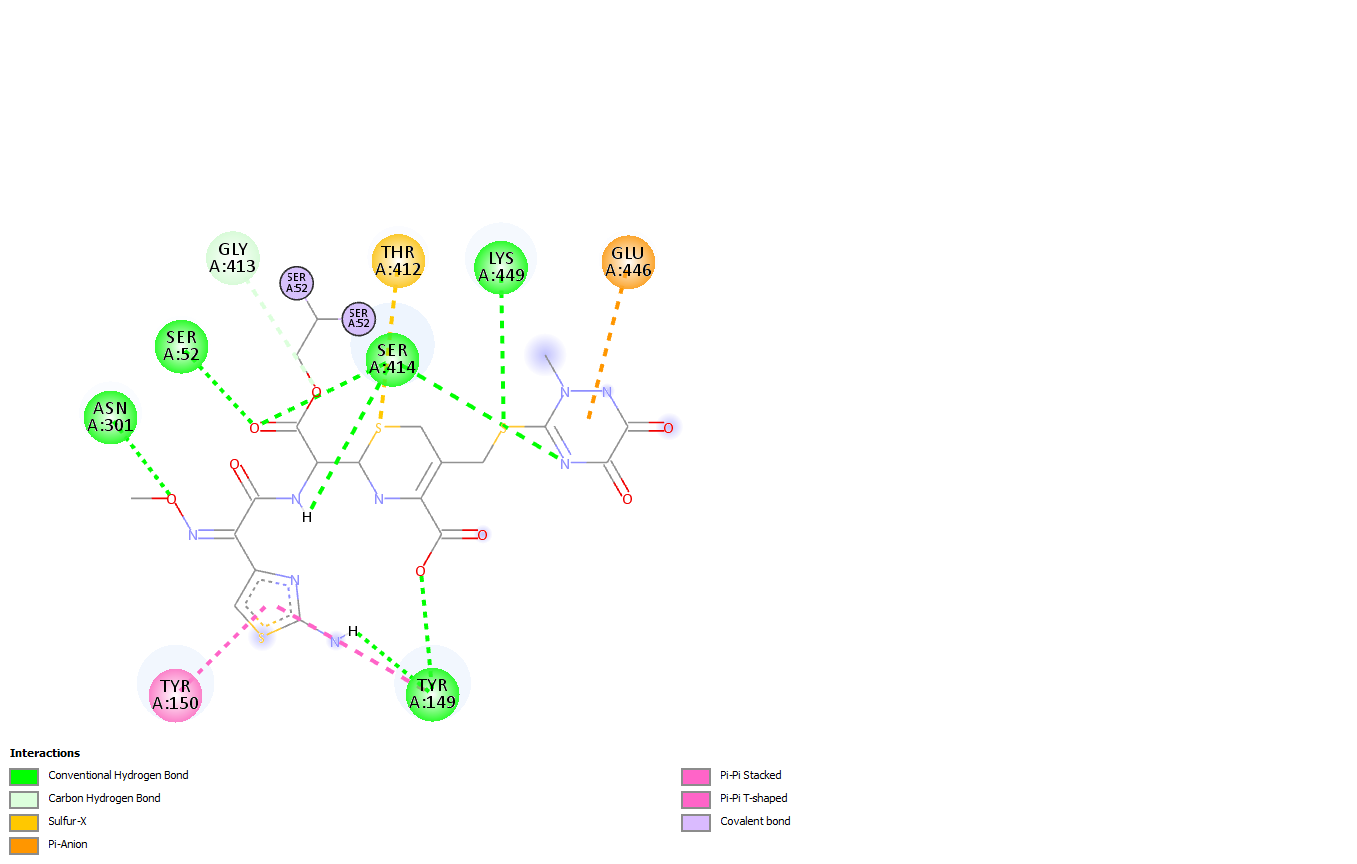 |
