# Supplementary material for: Synthesis and biological evaluation of the progenitor of a new class of cephalosporin analogues, with a particular focus on structure-based computational analysis
Source: PLoS One. 2017 Jul 27;12(7):e0181563. doi: 10.1371/journal.pone.0181563 (PMC5531512; doi:10.1371/journal.pone.0181563)
Supplement: S3 Table — In this table are reported for each protein the interactions of the representative pose(s) reported in Table 4. See the legend below the scheme to understand the different interactions. (DOCX) [file pone.0181563.s003.docx]

**S3 Table: details of the interactions between compound 8, ceftriaxone and each PBP from Gram negative bacteria.**

In this table are reported for each protein the interactions of the representative pose(s) reported in Table 4. See the legend below the scheme to understand the different interactions.

**5FGZ (PBP1b from *E. coli*)**

| **Ligand** | **ΔG (kcal/mol)** | **Scheme** |
| --- | --- | --- |
| compound 8 (3R4S), ring A reactive | -11.23 | 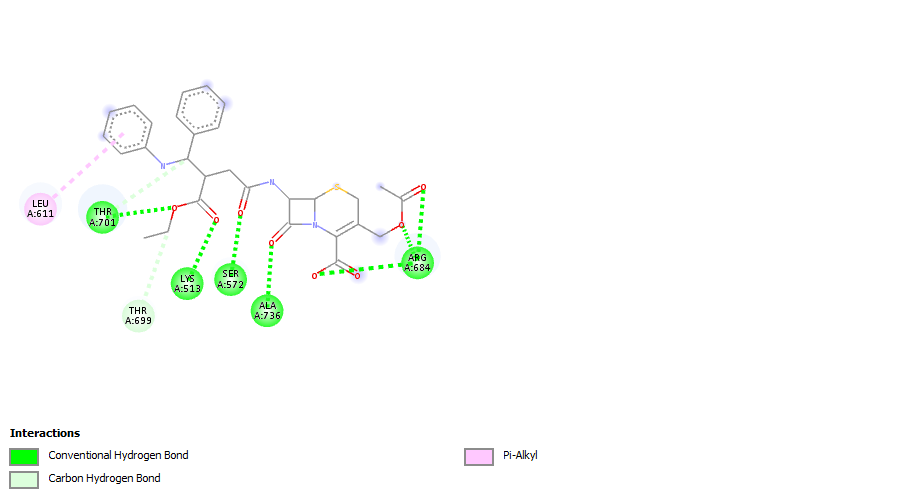 |
|  | -10.48 | 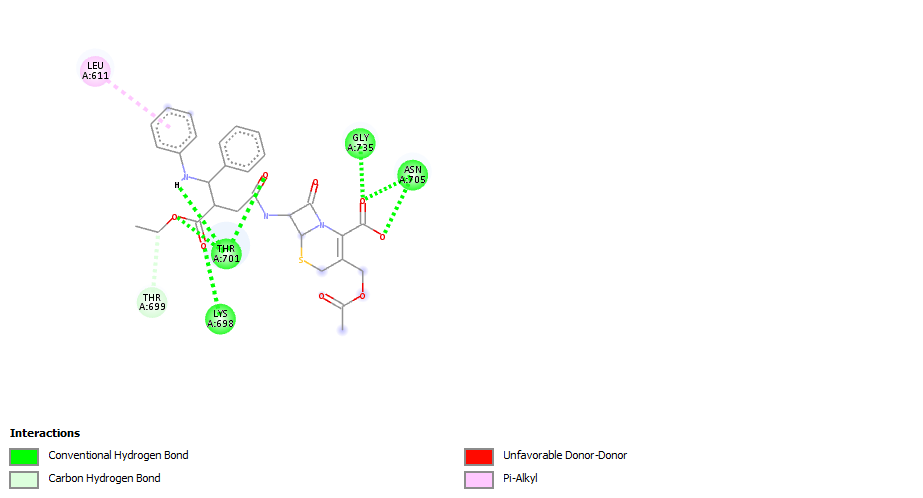 |
| compound 8 (3R4S), ring B reactive | -13.02 | 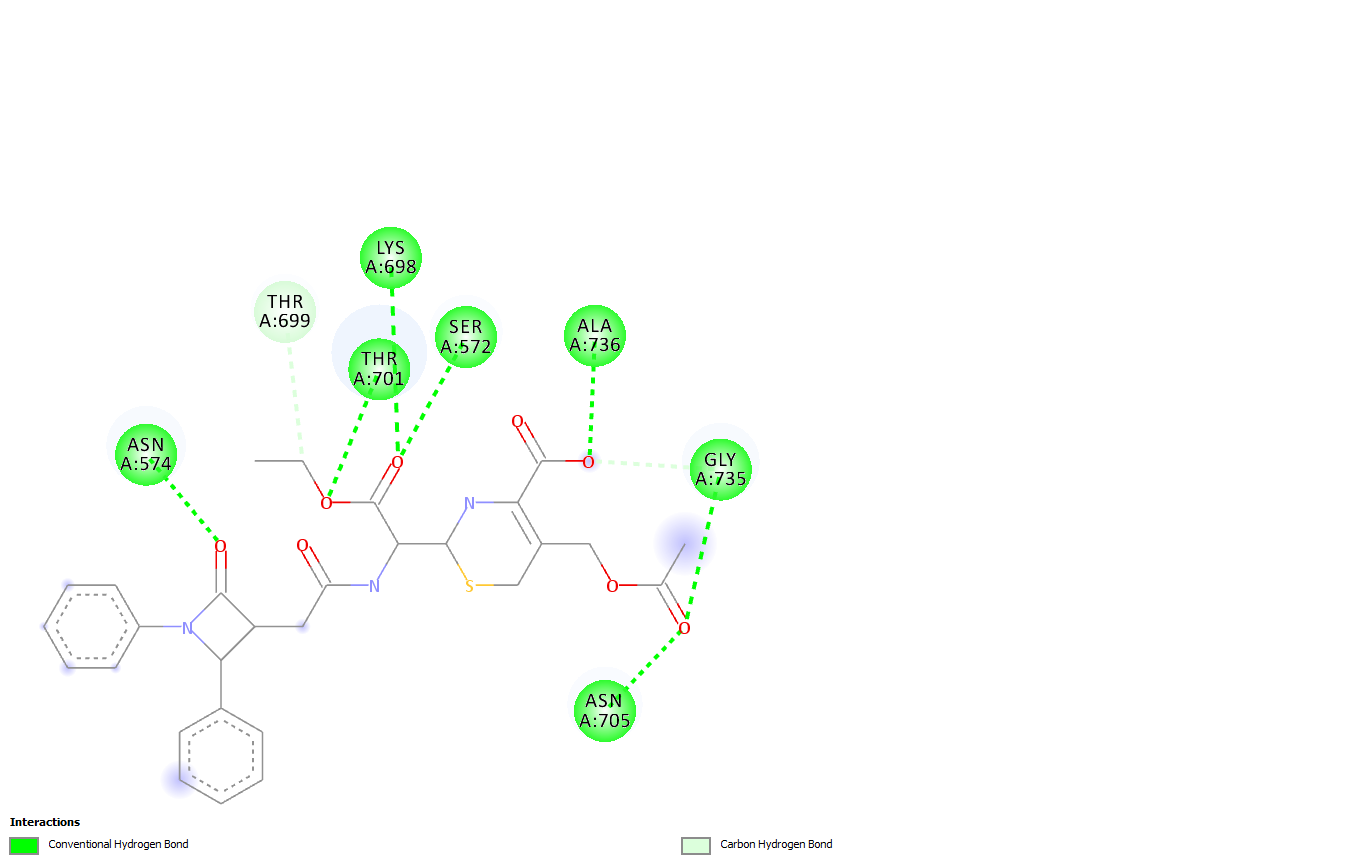 |
| compound 8 (3S4R), ring A reactive | -11.29 | 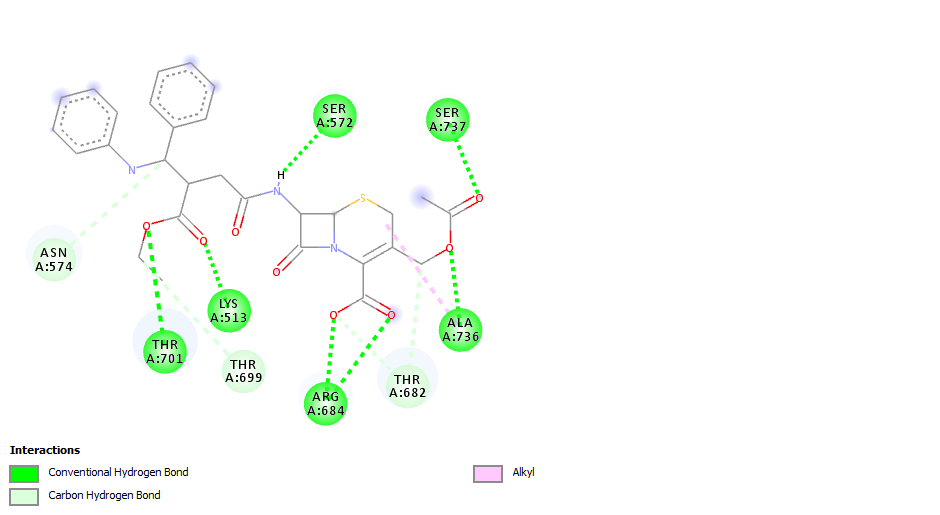 |
|  | -11.23 | 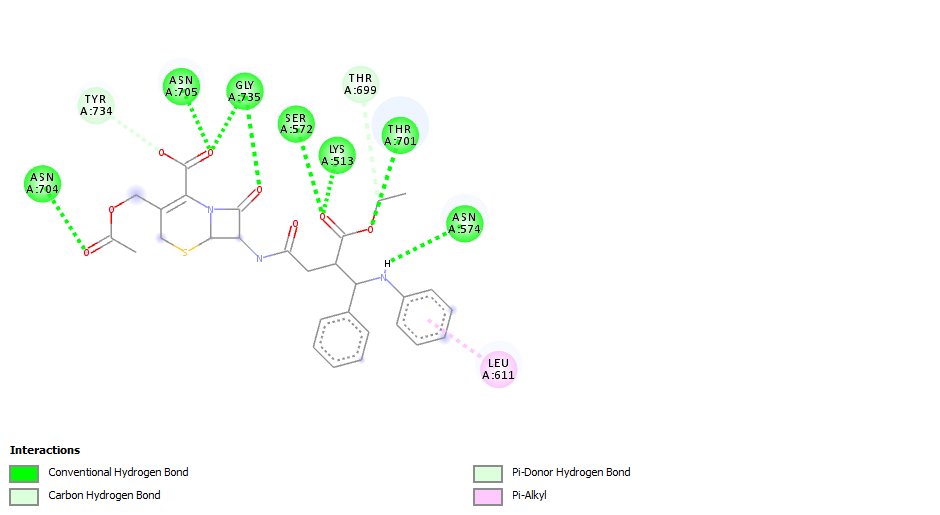 |
| compound 8 (3S4R), ring B reactive | -12.73 | 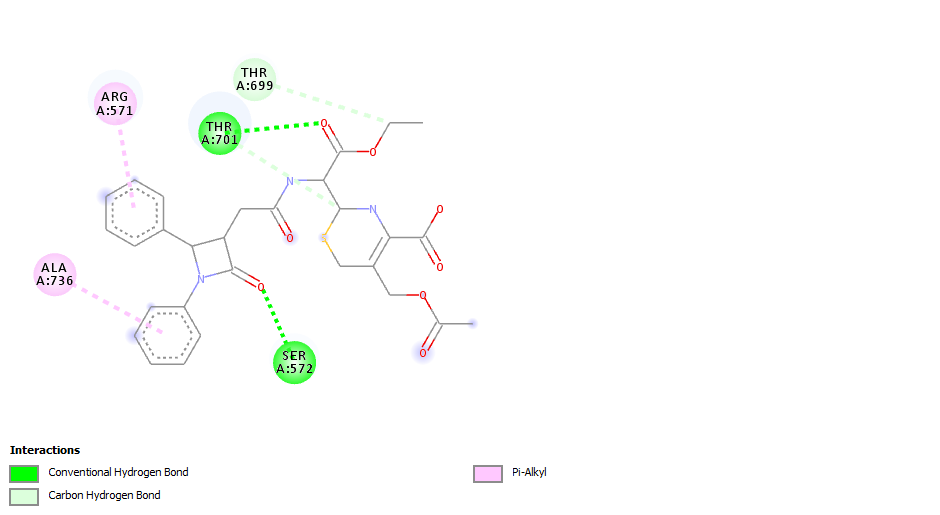 |
|  | -12.42 | 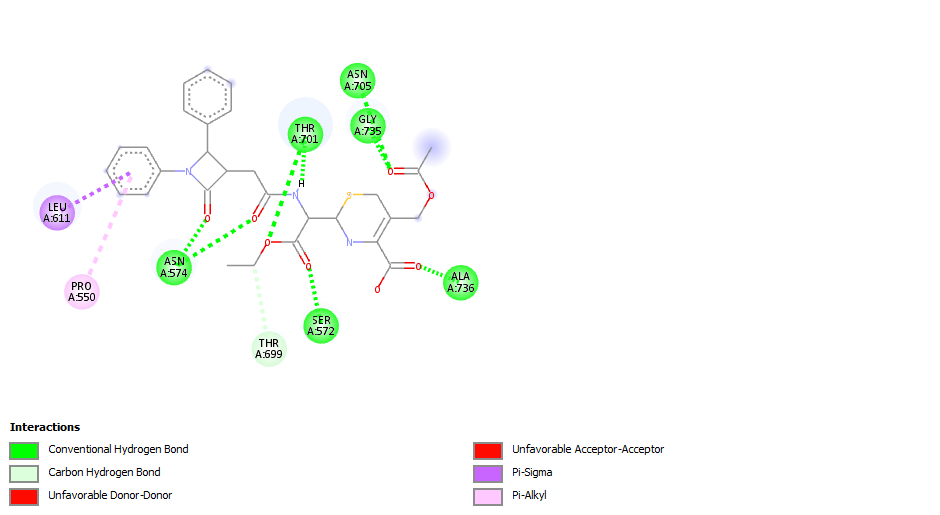 |
| Ceftriaxone | -11.87 | 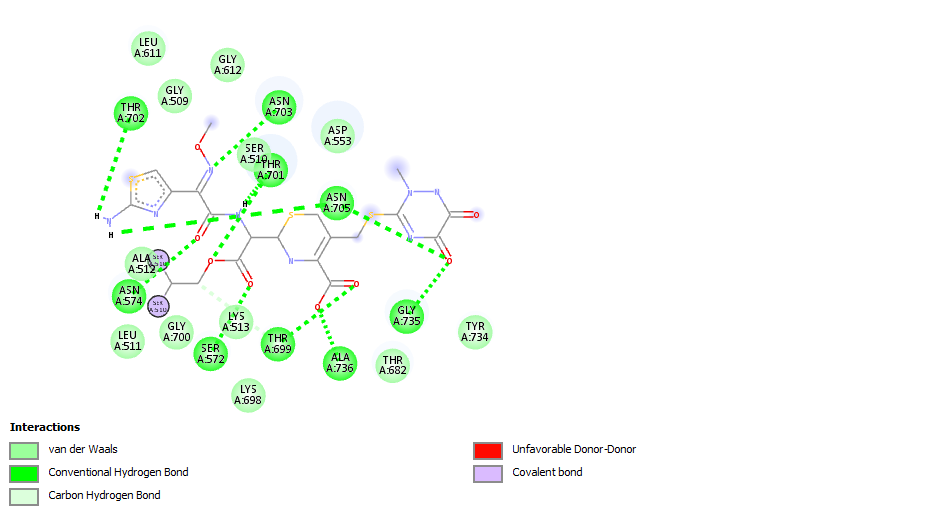 |
|  | -11.39 | 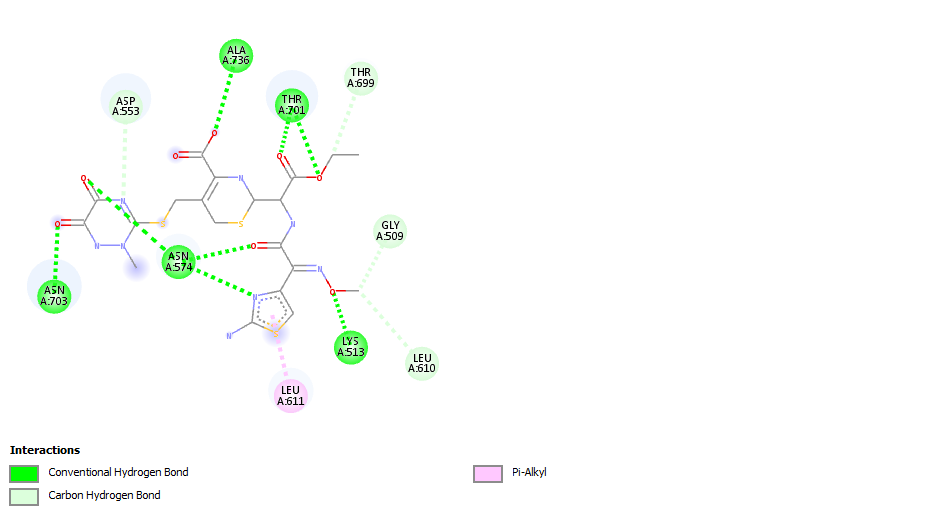 |

**4BJP (PBP3 from E. coli) (S3 Table – cont.)**

| **Ligand** | **ΔG (kcal/mol)** | **Scheme** |
| --- | --- | --- |
| compound 8 (3R4S), ring A reactive | -11.17 | 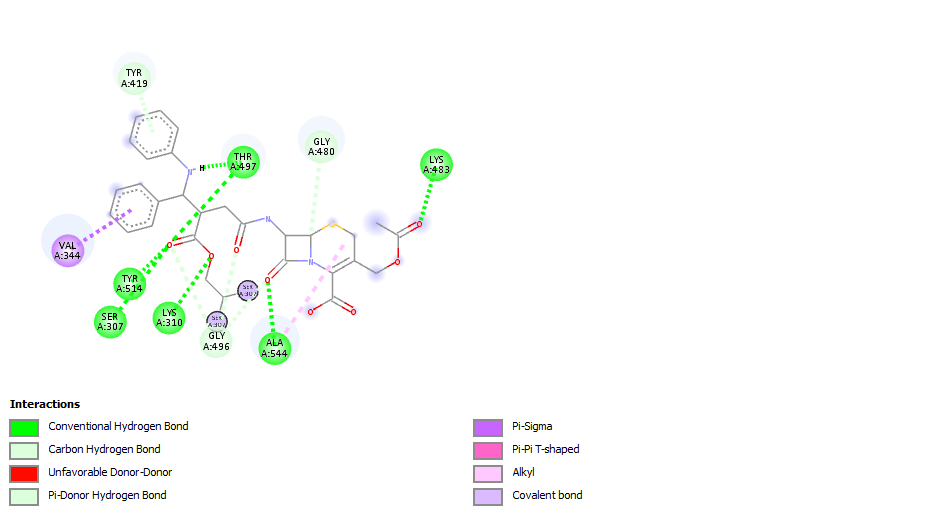 |
| compound 8 (3R4S), ring B reactive | -13.11 | 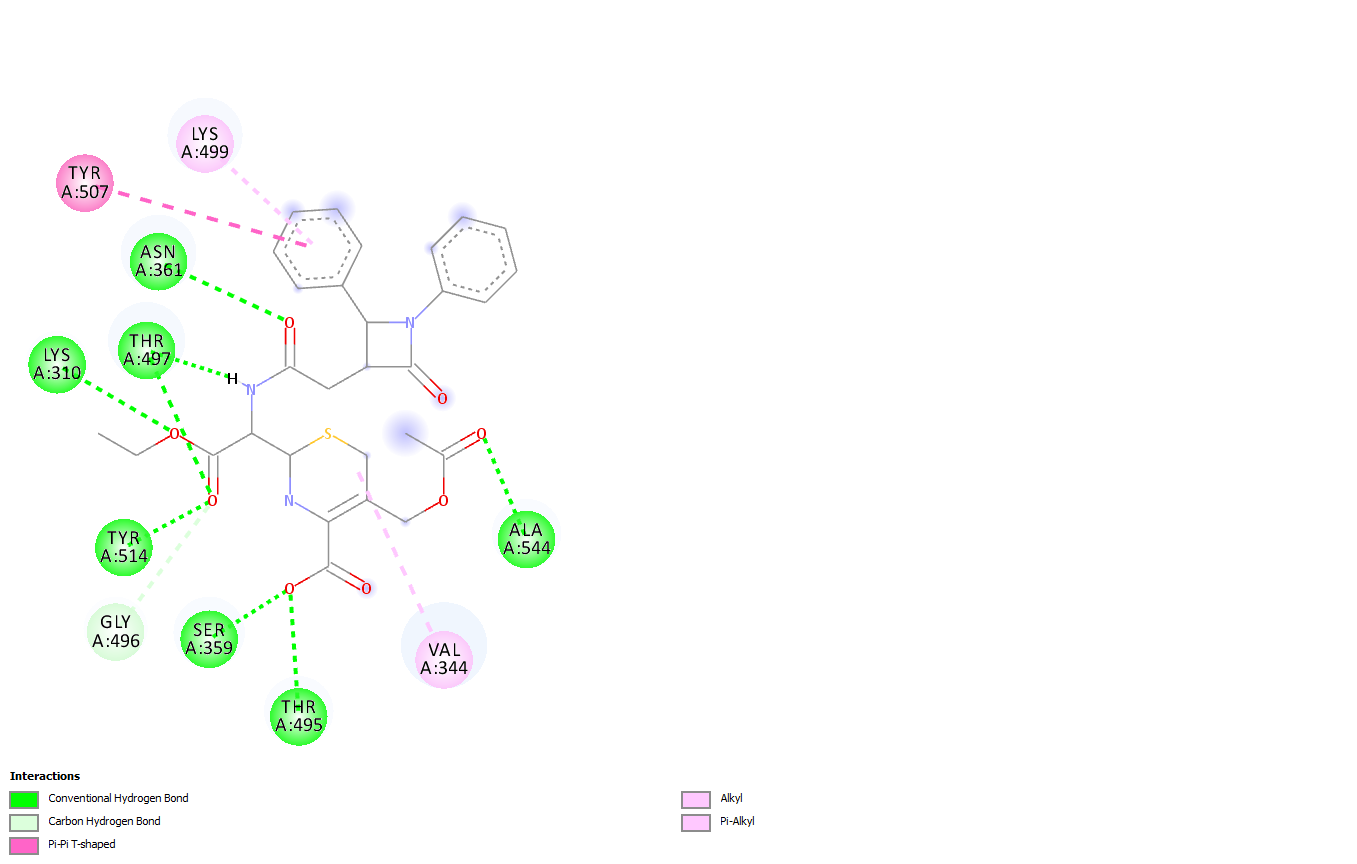 |
| compound 8 (3S4R), ring A reactive | -11.83 | 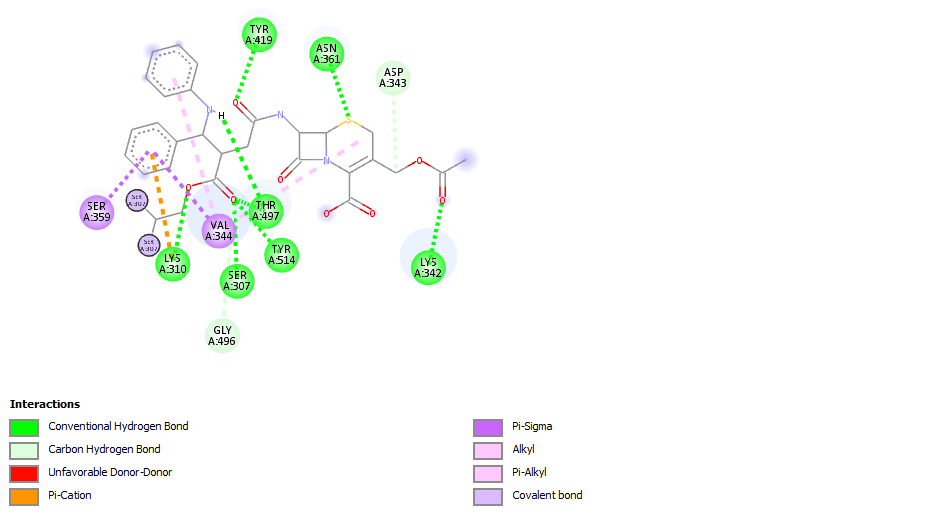 |
| compound 8 (3S4R), ring B reactive | -12.48 | 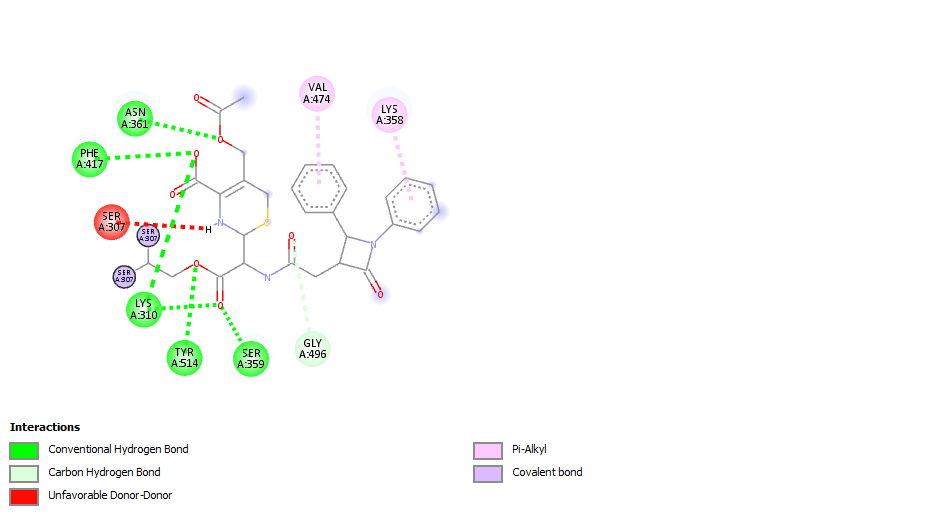 |
| Ceftriaxone | -12.55 | 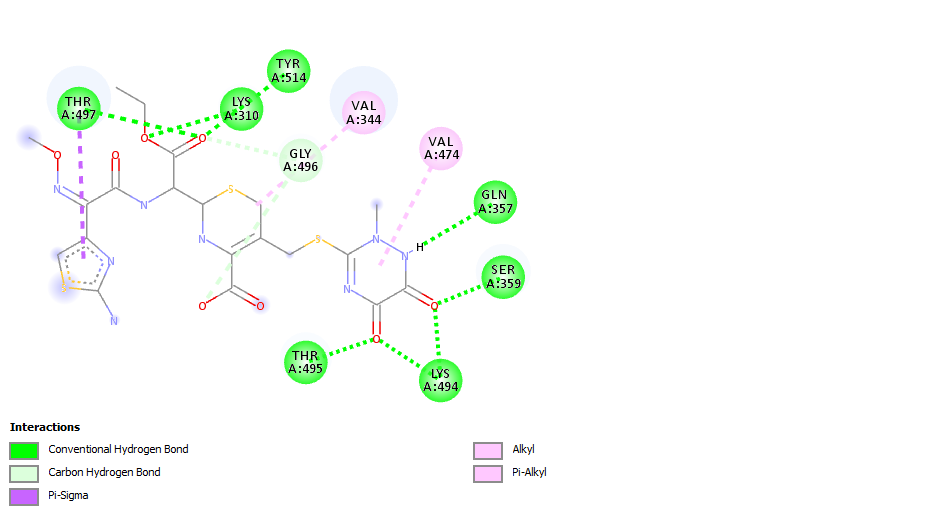 |
|  | -12.21 | 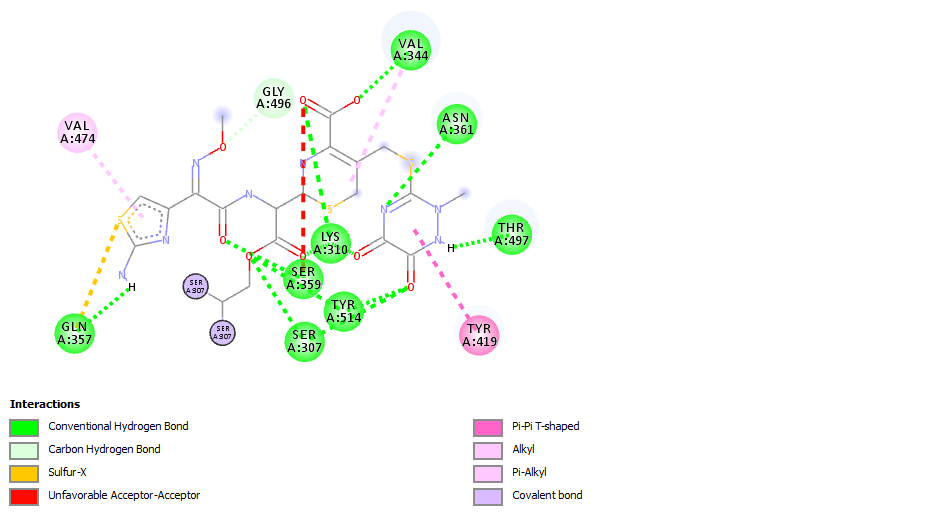 |

**2EX8 (PBP4 from *E. coli*) (Suppl. Table 3 – cont.)**

| **Ligand** | **ΔG (kcal/mol)** | **Scheme** |
| --- | --- | --- |
| compound 8 (3R4S), ring A reactive | -11.76 | 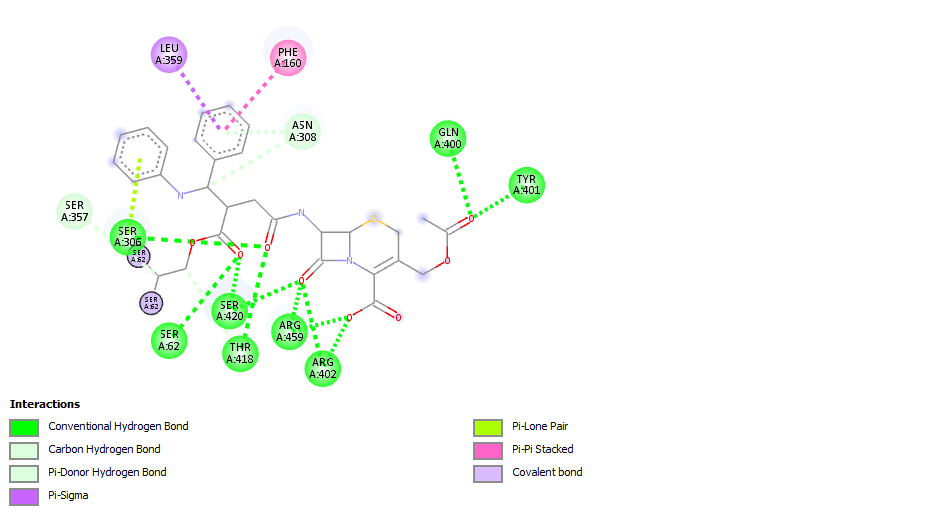 |
| compound 8 (3R4S), ring B reactive | -12.14 | 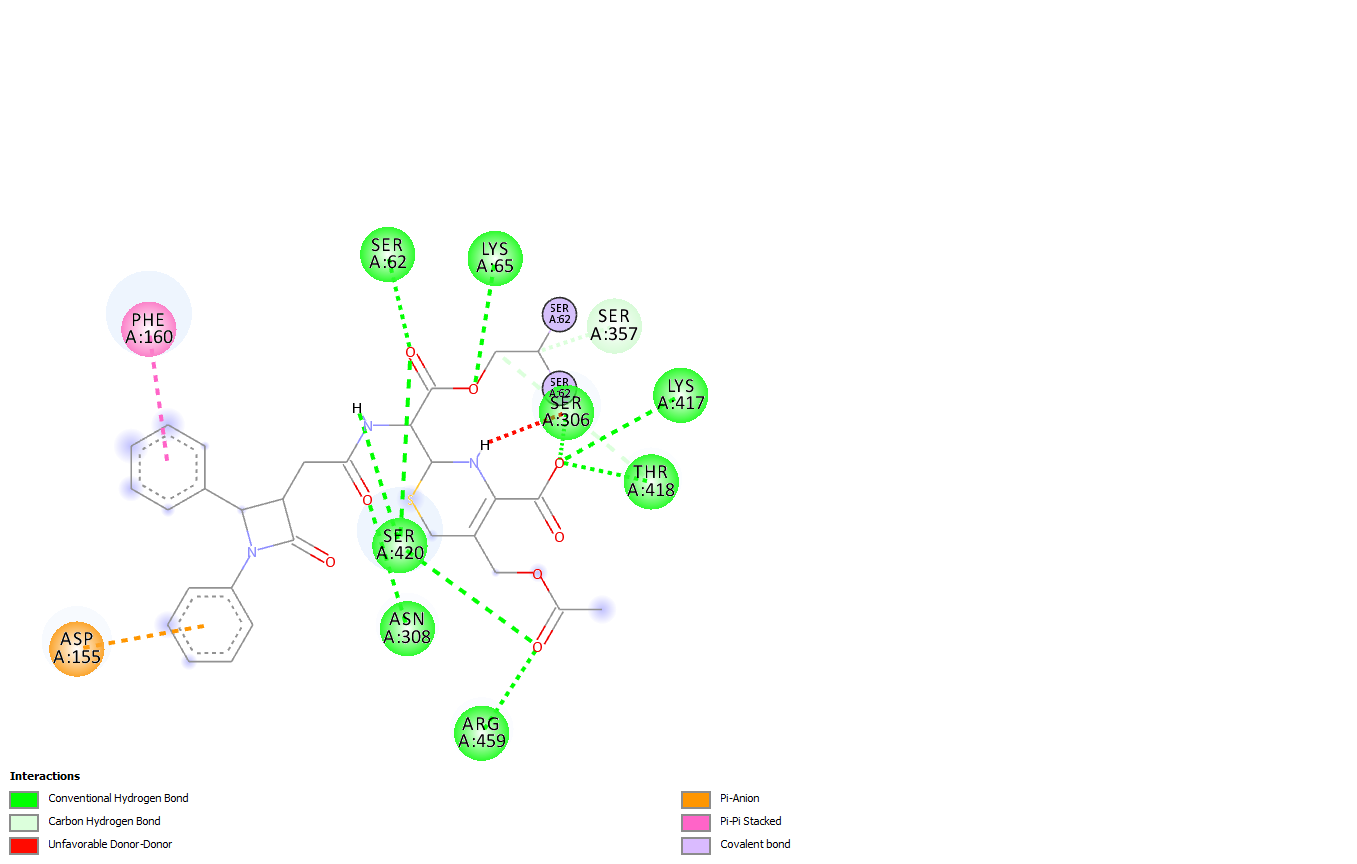 |
|  | -11.95 | 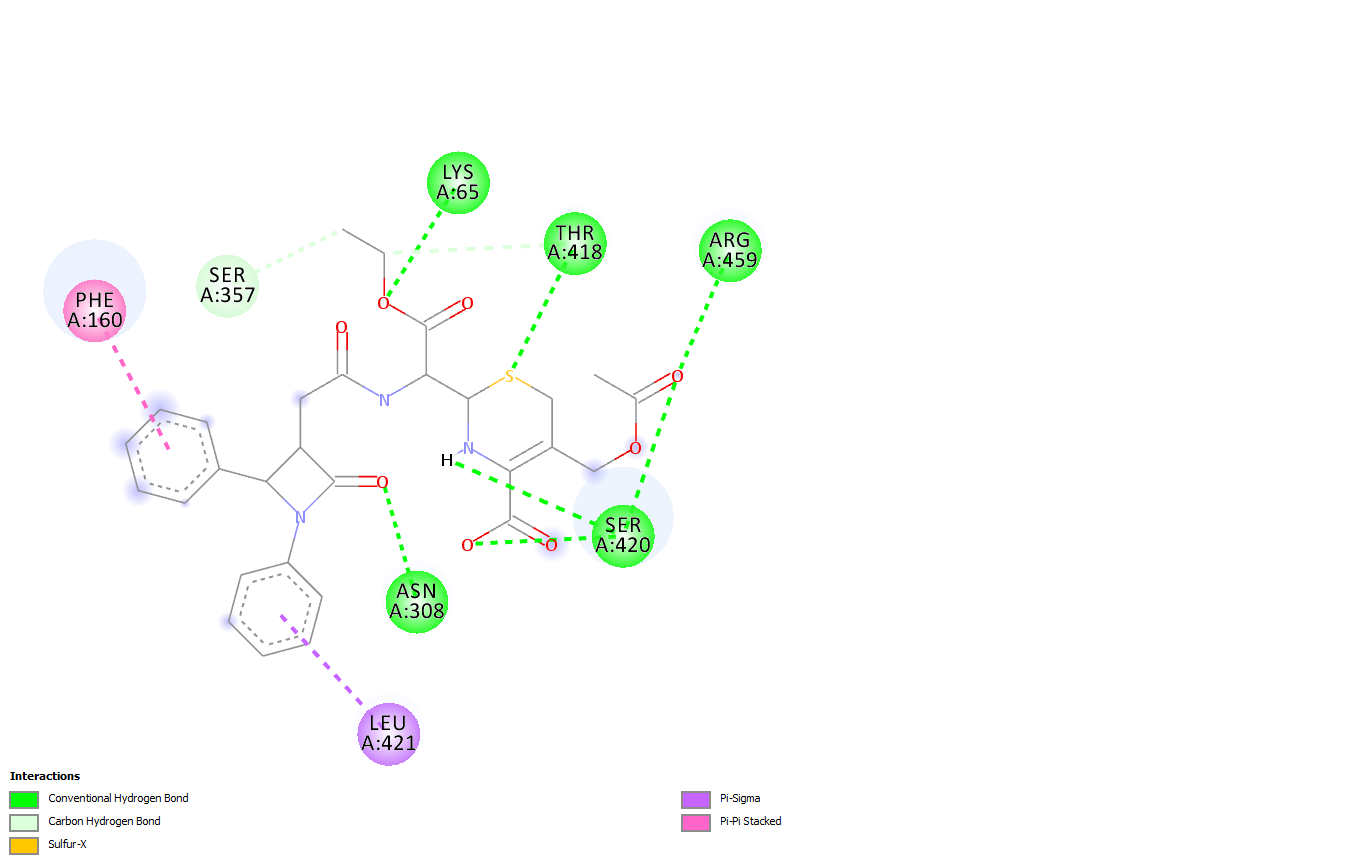 |
|  | -11.92 | 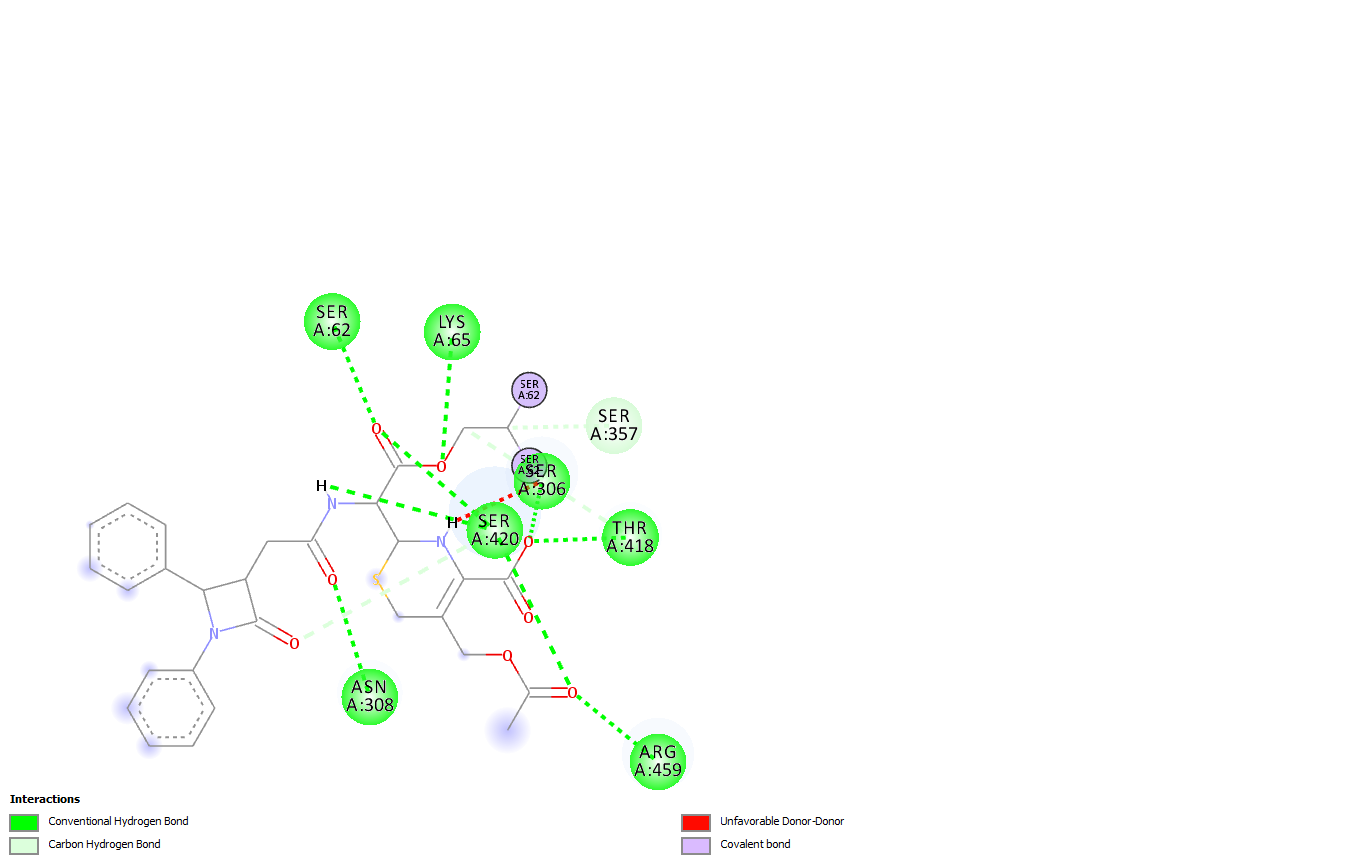 |
| compound 8 (3S4R), ring A reactive | -10.15 | 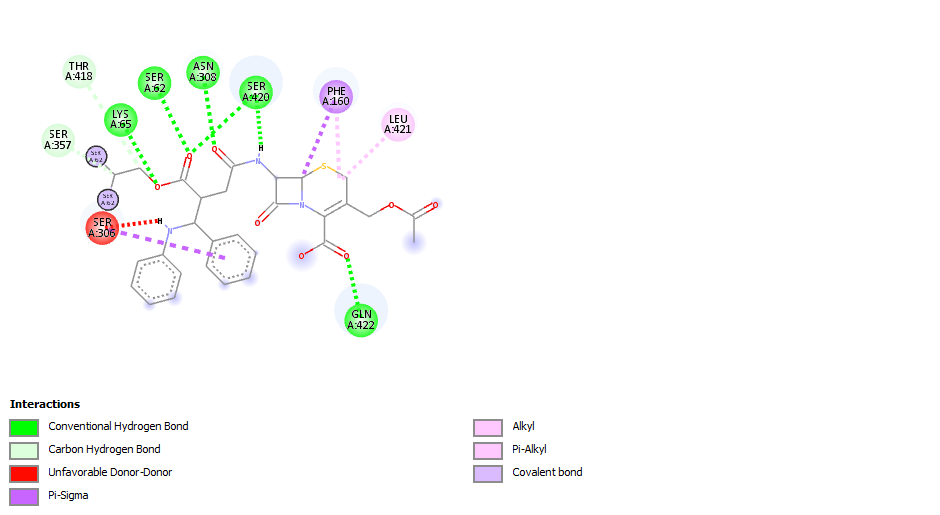 |
| compound 8 (3S4R), ring B reactive | -13.46 | 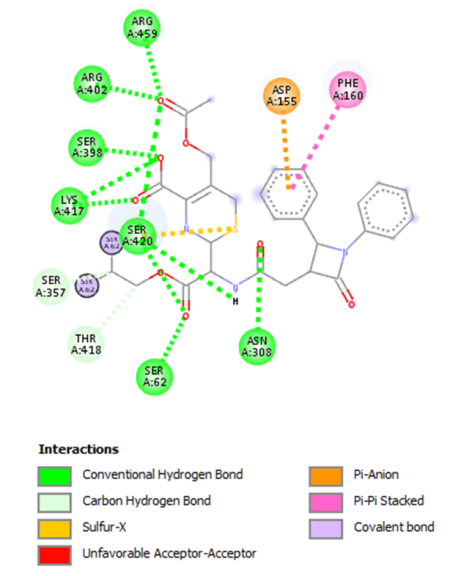 |
| Ceftriaxone | -12.76 | 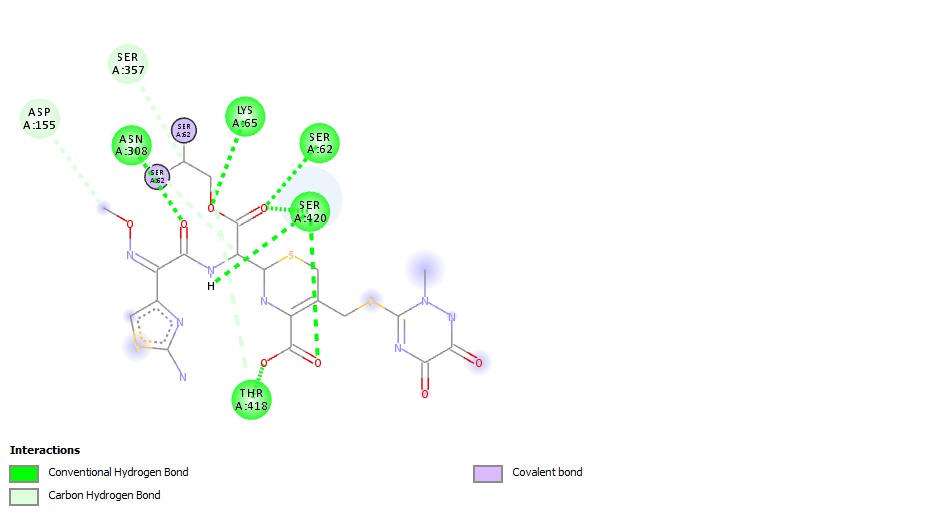 |
|  | -11.77 | 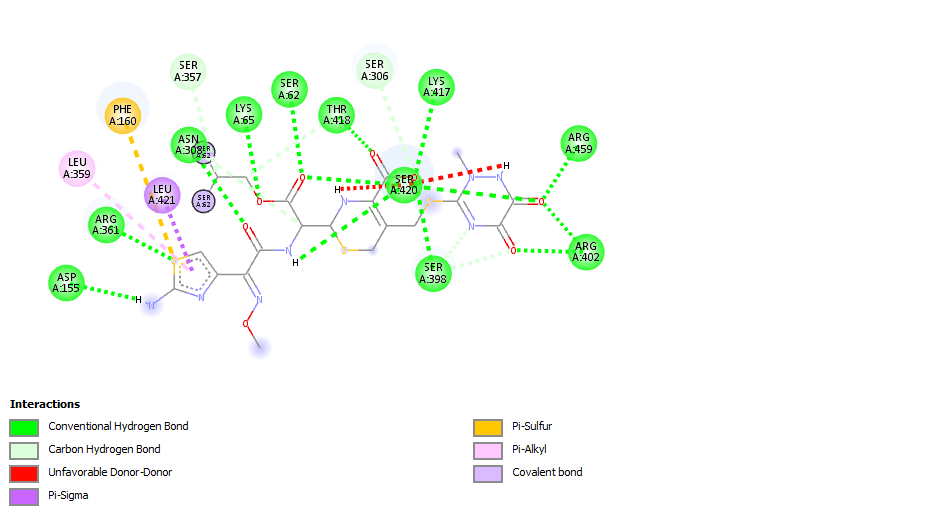 |

**1Z6F (PBP5 from *E. coli*) (S3. Table – cont.)**

| **Ligand** | **ΔG (kcal/mol)** | **Scheme** |
| --- | --- | --- |
| compound 8 (3R4S), ring A reactive | -10.25 | 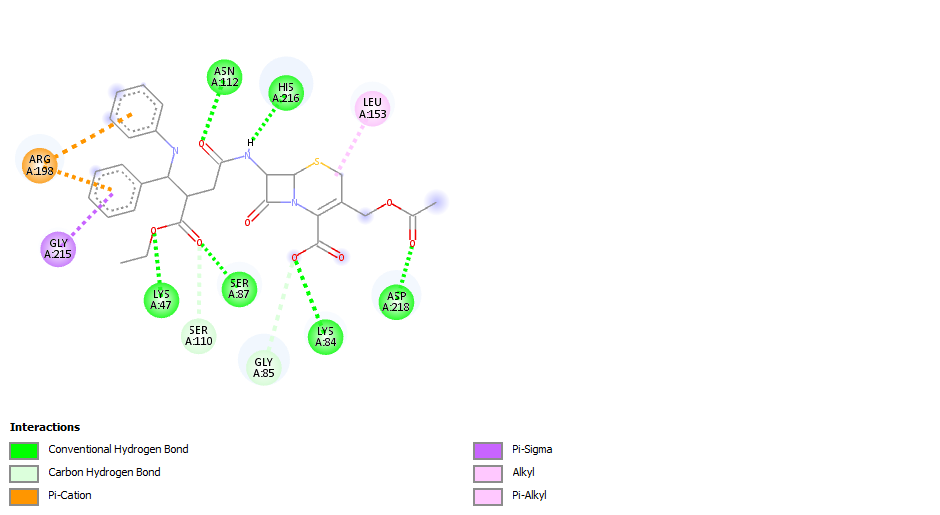 |
| compound 8 (3R4S), ring B reactive | -12.73 | 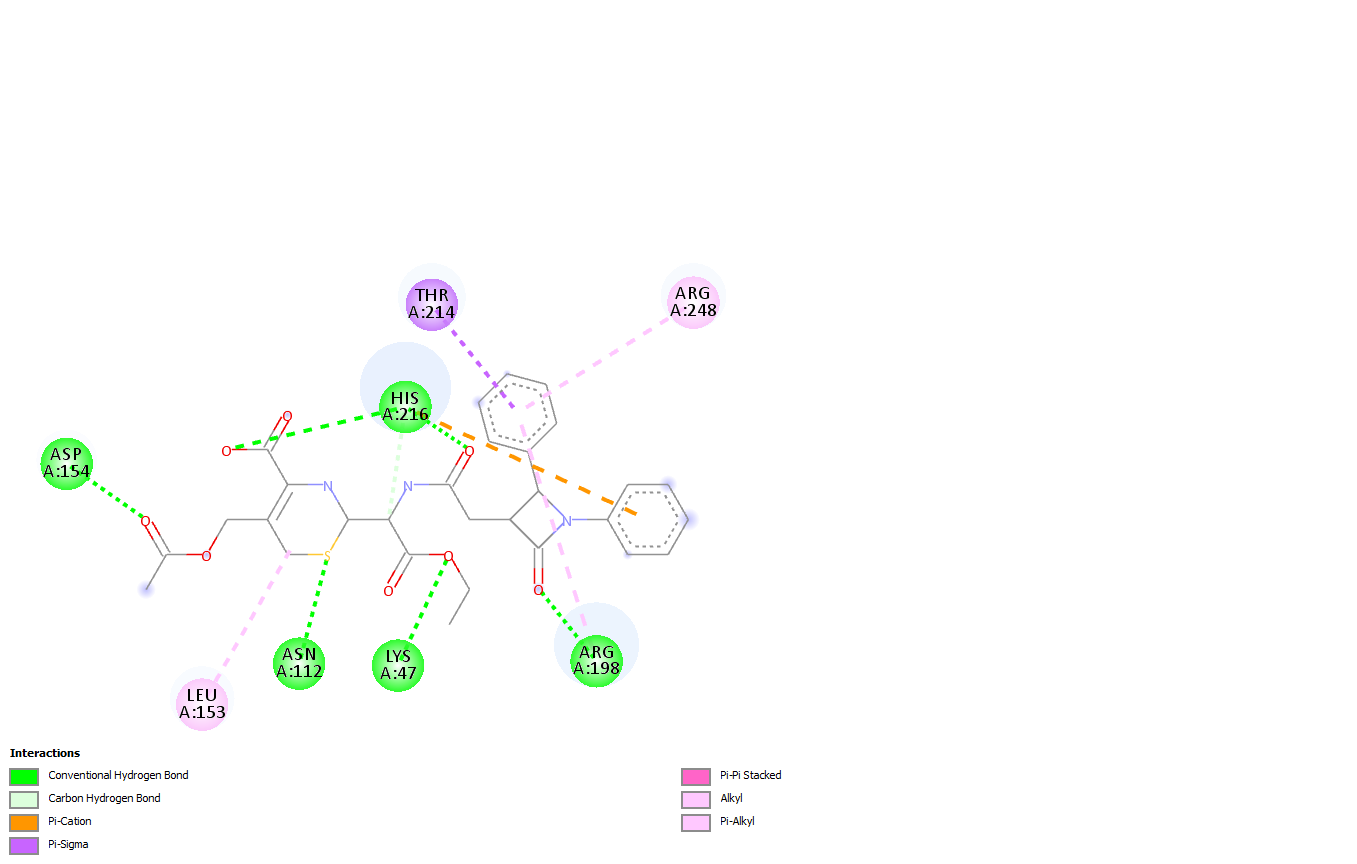 |
| compound 8 (3S4R), ring A reactive | -9.84 | 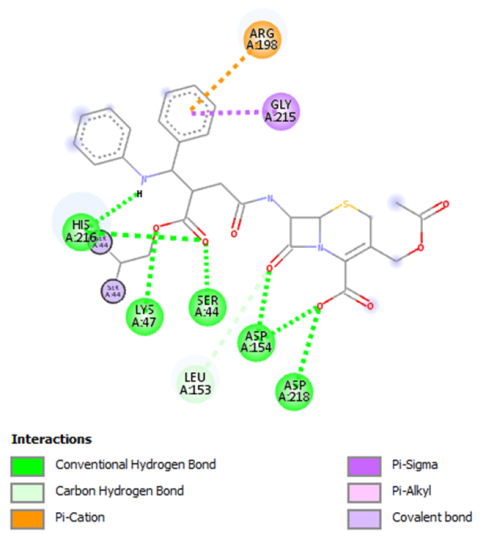 |
| compound 8 (3S4R), ring B reactive | -11.58 | 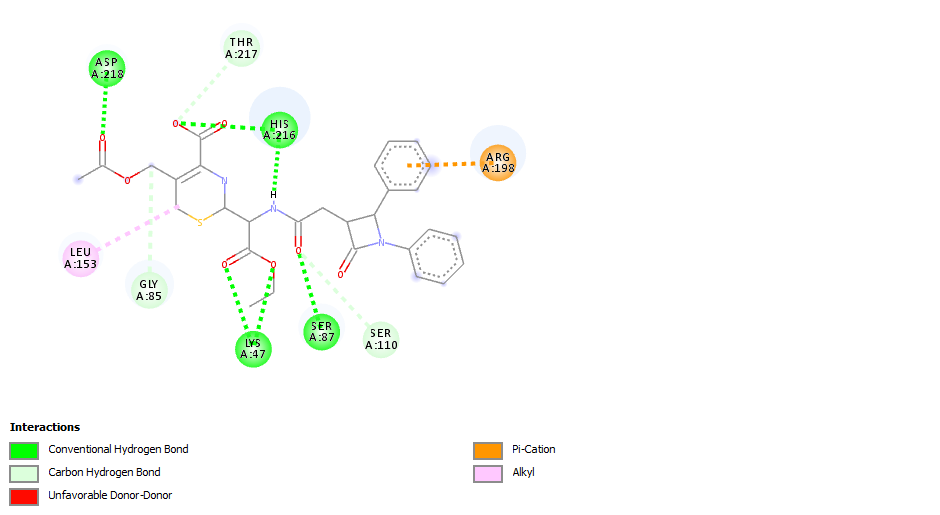 |
| Ceftriaxone | -11.33 | 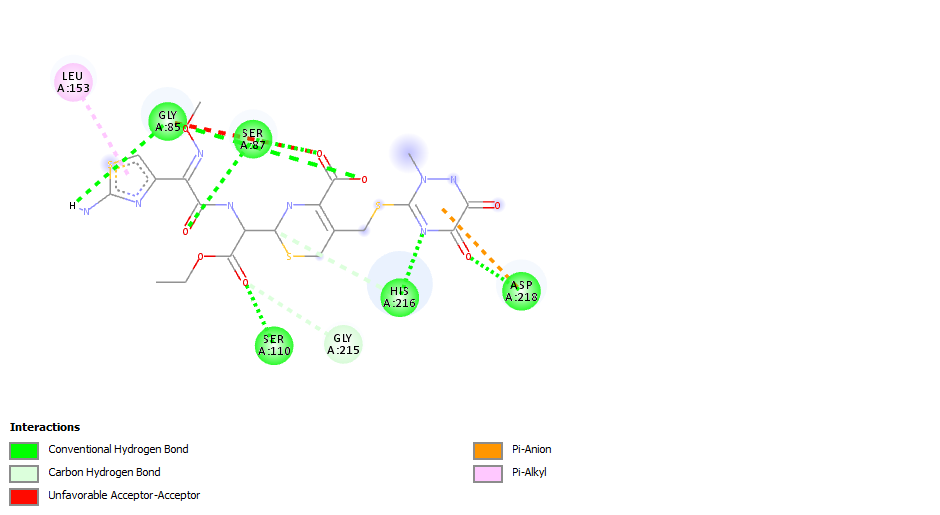 |

**3OCL (PBP3 from *P. aeruginosa*) (S3 Table – cont.)**

| **Ligand** | **ΔG (kcal/mol)** | **Scheme** |
| --- | --- | --- |
| compound 8 (3R4S), ring A reactive | -11.62 | 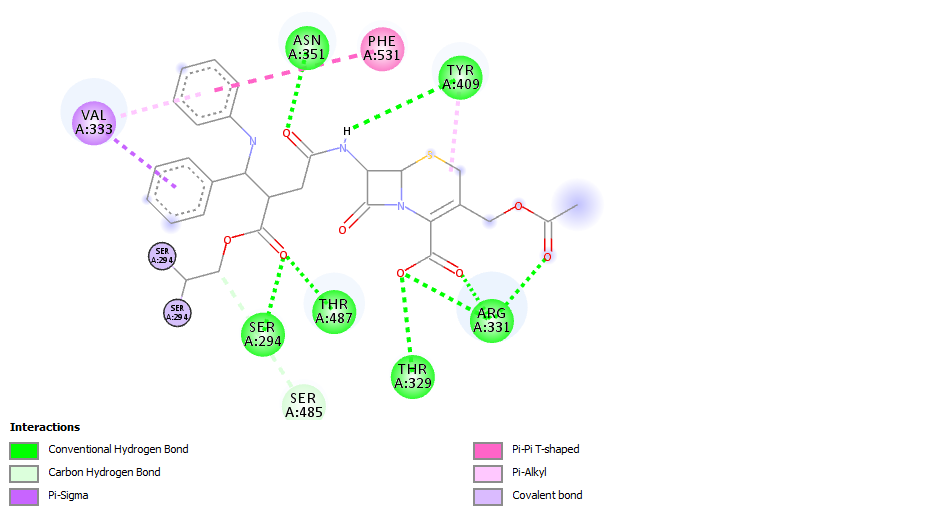 |
| compound 8 (3R4S), ring B reactive | -14.30 | 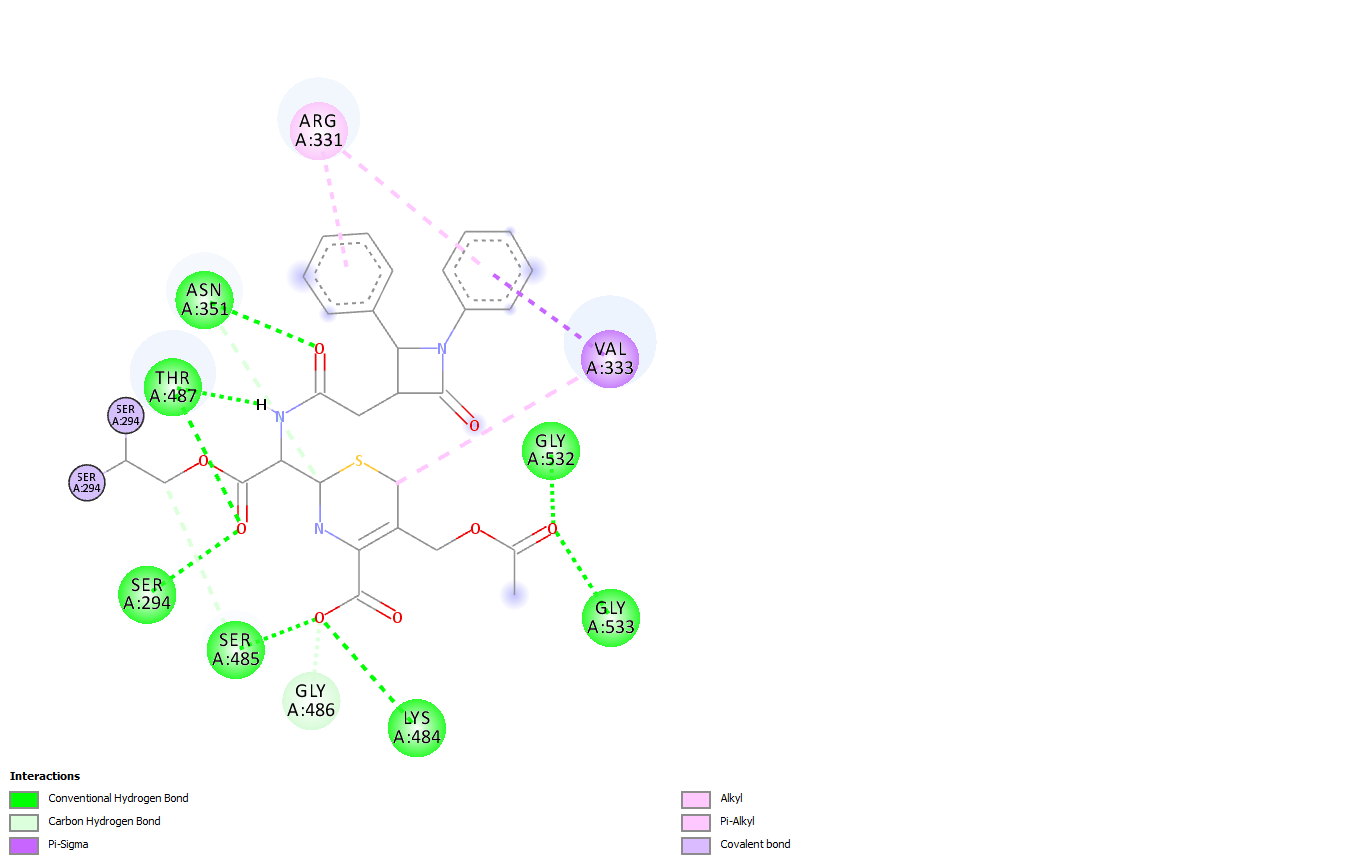 |
| compound 8 (3S4R), ring A reactive | -12.29 | 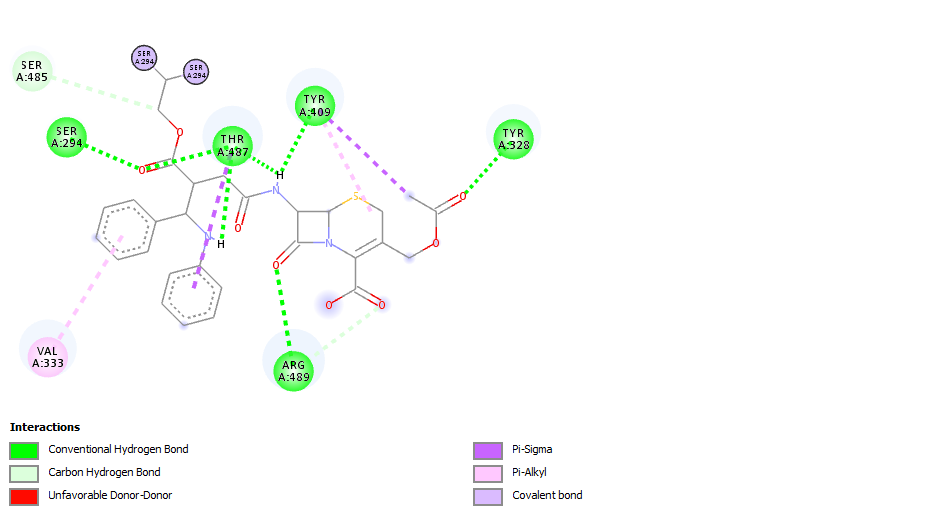 |
| compound 8 (3S4R), ring B reactive | -14.16 | 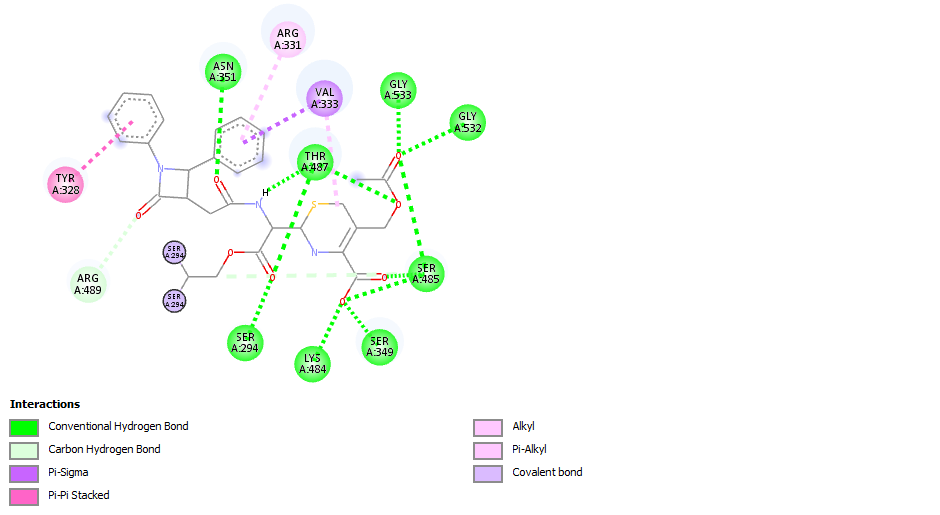 |
| Ceftriaxone | -13.99 | 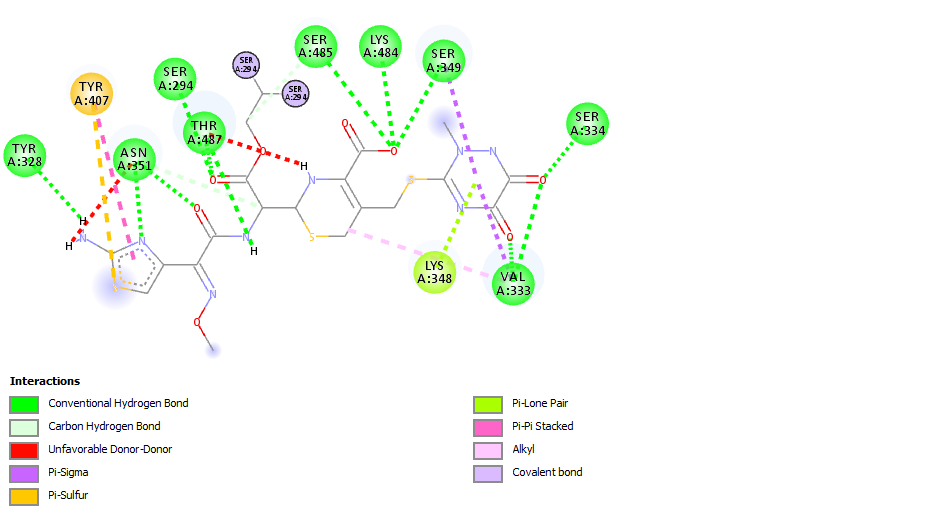 |
|  | -11.98 | 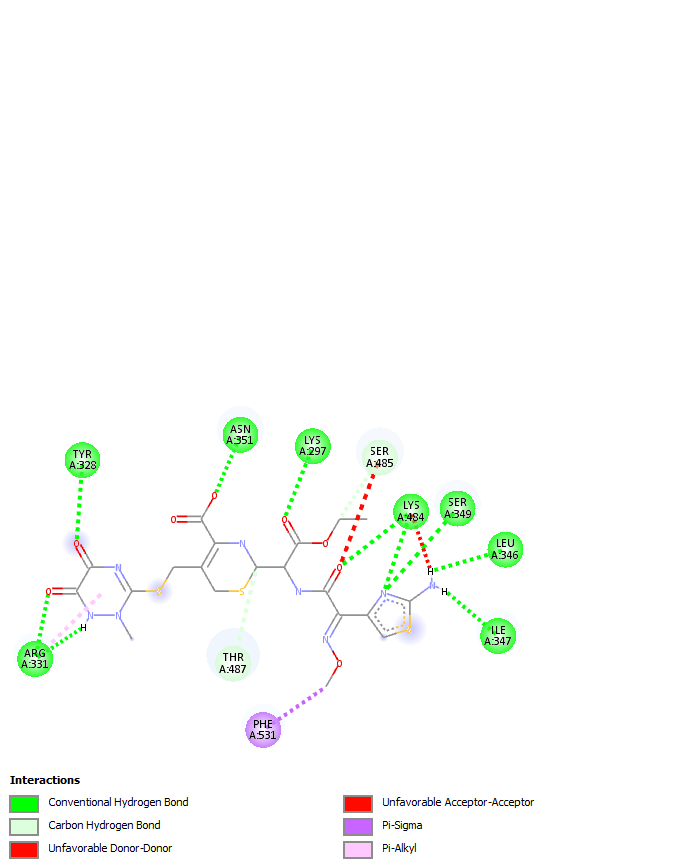 |

**4K91 (PBP5 from *P. aeruginosa*) (S3 Table – cont.)**

| **Ligand** | **ΔG (kcal/mol)** | **Scheme** |
| --- | --- | --- |
| compound 8 (3R4S), ring A reactive | -10.09 | 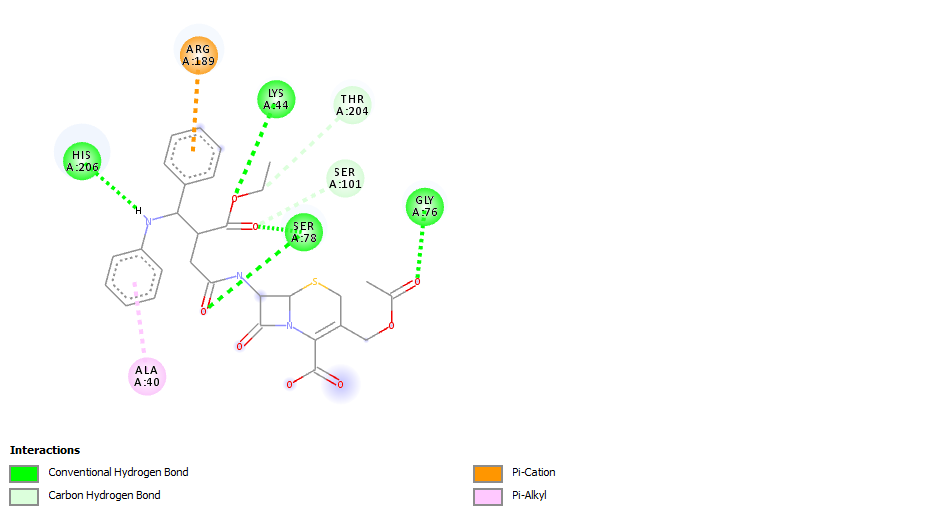 |
|  | -9.41 | 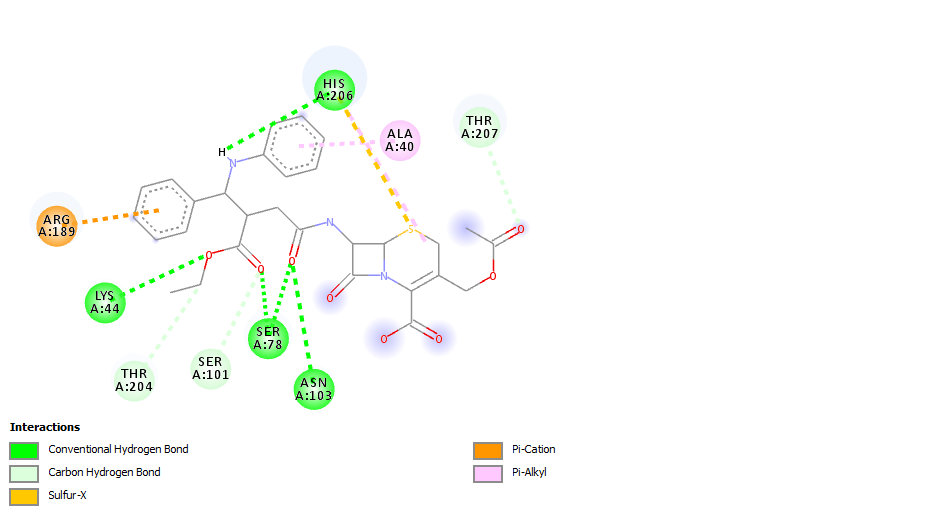 |
| compound 8 (3R4S), ring B reactive | -12.86 | 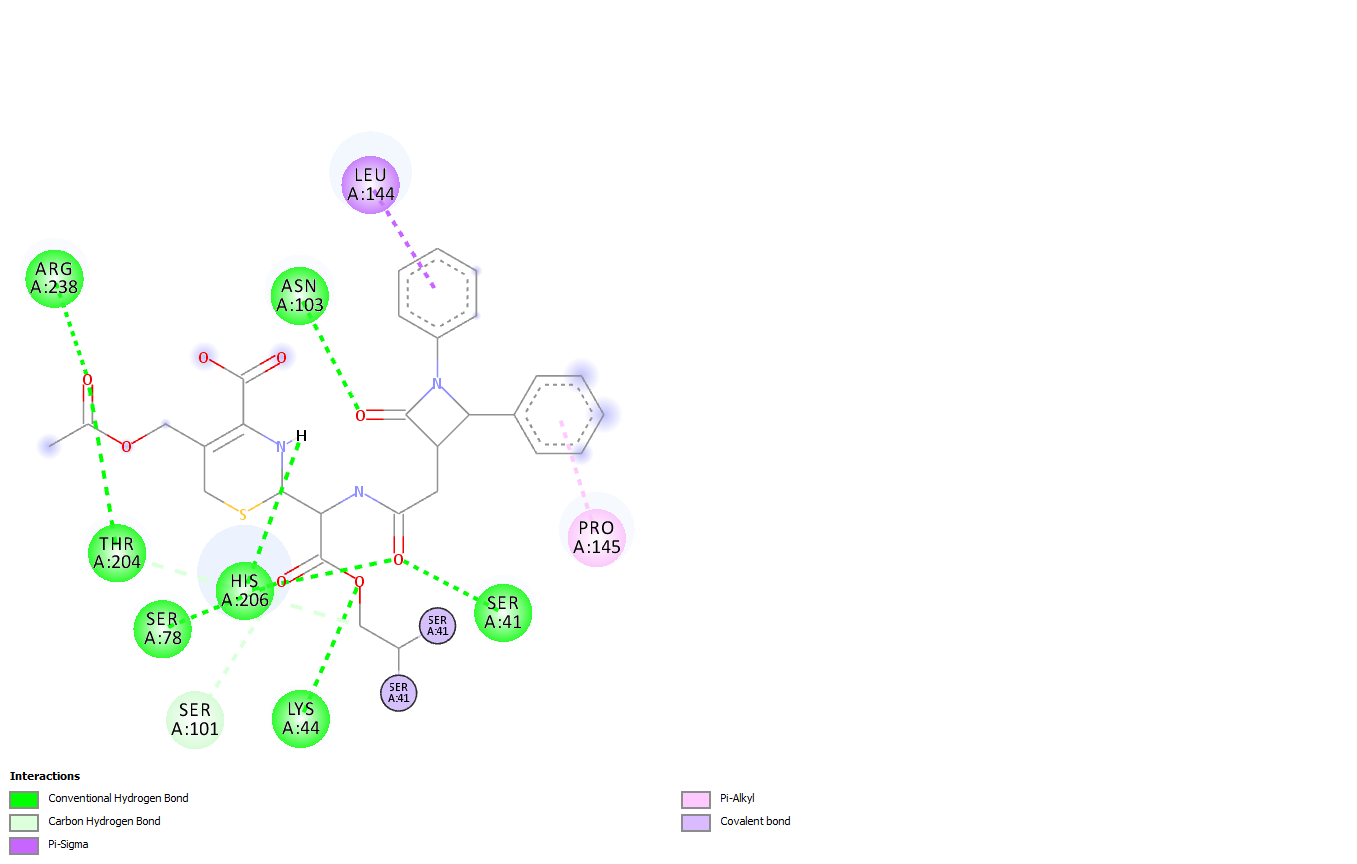 |
| compound 8 (3S4R), ring A reactive | -10.18 | 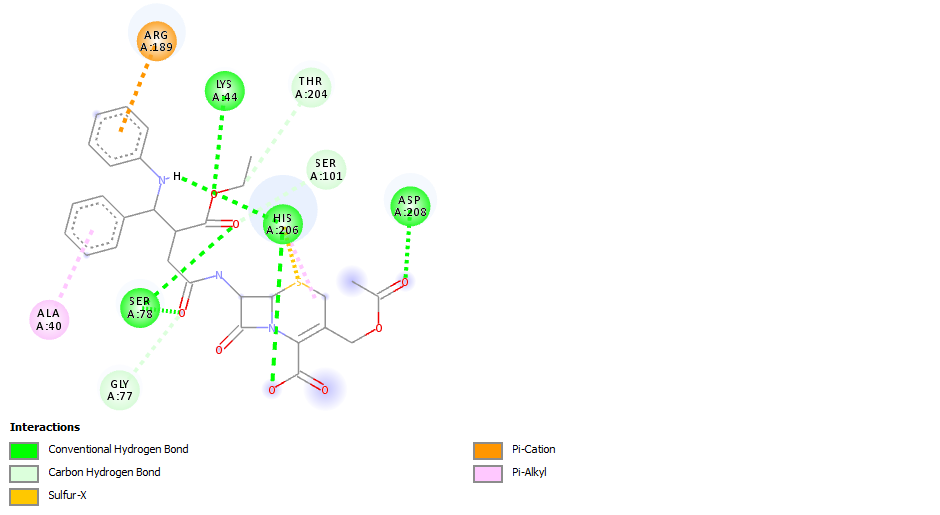 |
|  | -9.69 | 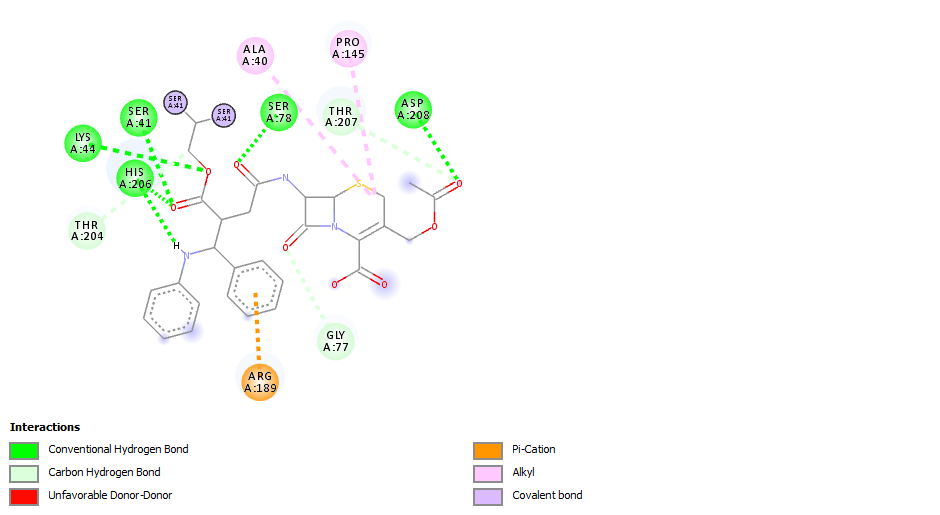 |
| compound 8 (3S4R), ring B reactive | -13.55 | 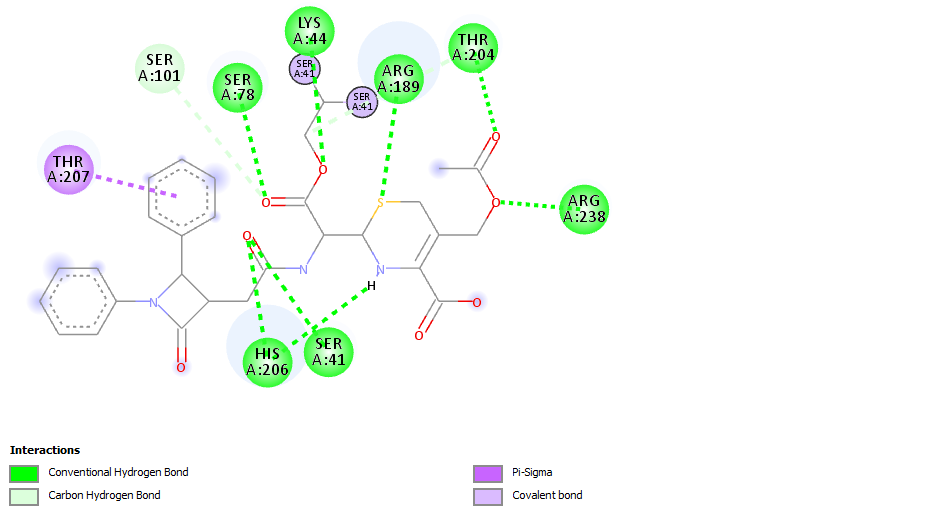 |
| Ceftriaxone | -12.14 | 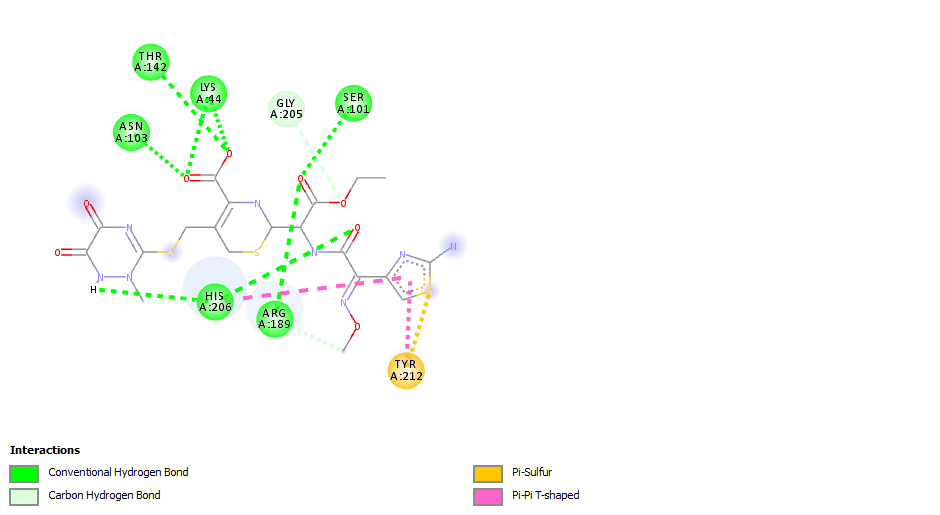 |
|  | -11.06 | 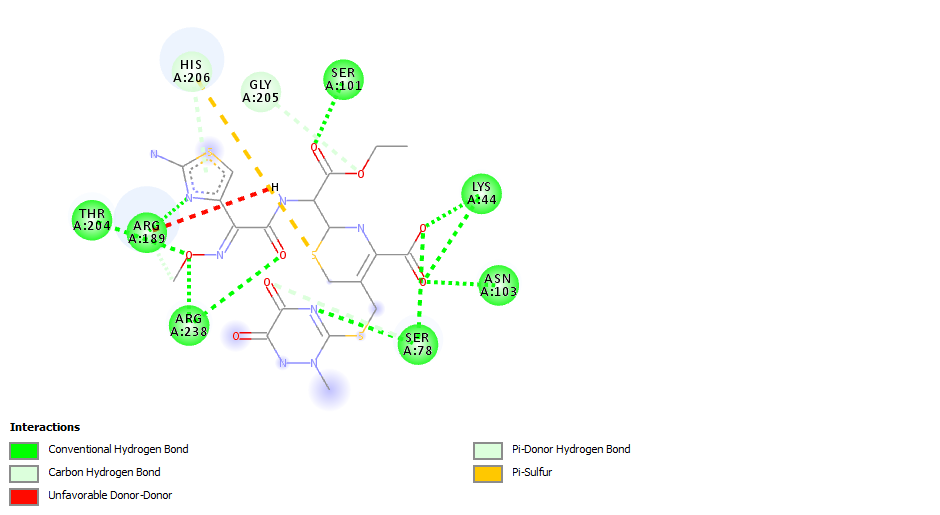 |
