# Supplementary figures and images for: Synthesis and biological evaluation of the progenitor of a new class of cephalosporin analogues, with a particular focus on structure-based computational analysis
Source: PLoS One. 2017 Jul 27;12(7):e0181563. doi: 10.1371/journal.pone.0181563 (PMC5531512; doi:10.1371/journal.pone.0181563)

**S1 Figure.** 1H NMR spectrum of compound **8** (600 MHz, D2O, 298 K).

**
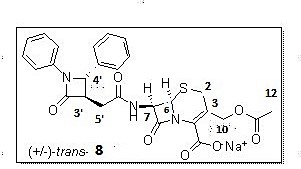

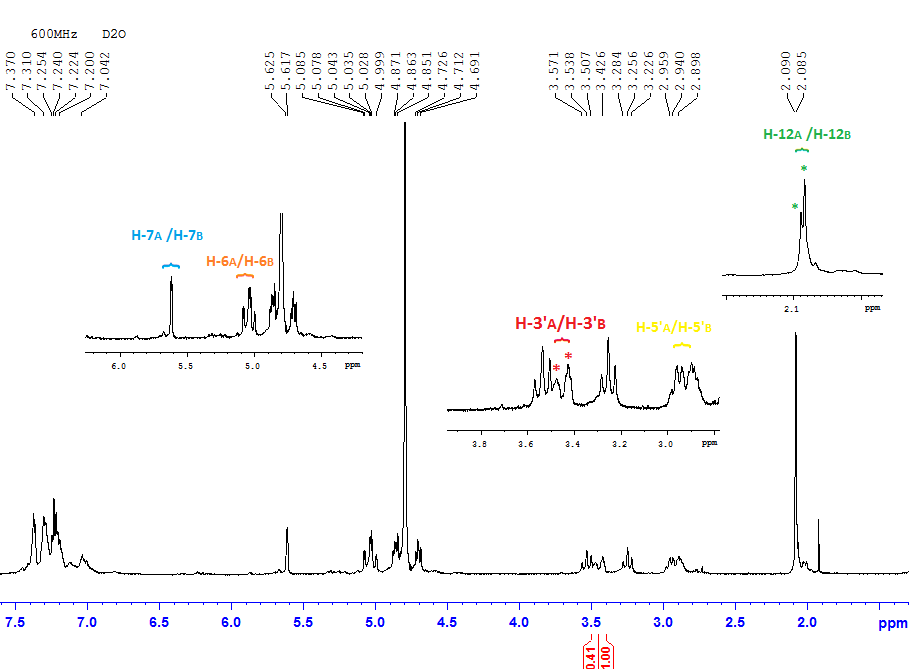
**

Supplement: S1 Fig — (DOCX) [file pone.0181563.s004.docx]

**S2 Figure.** 2D-COSY spectrum of compound **8** (600 MHz, D2O, 298 K).
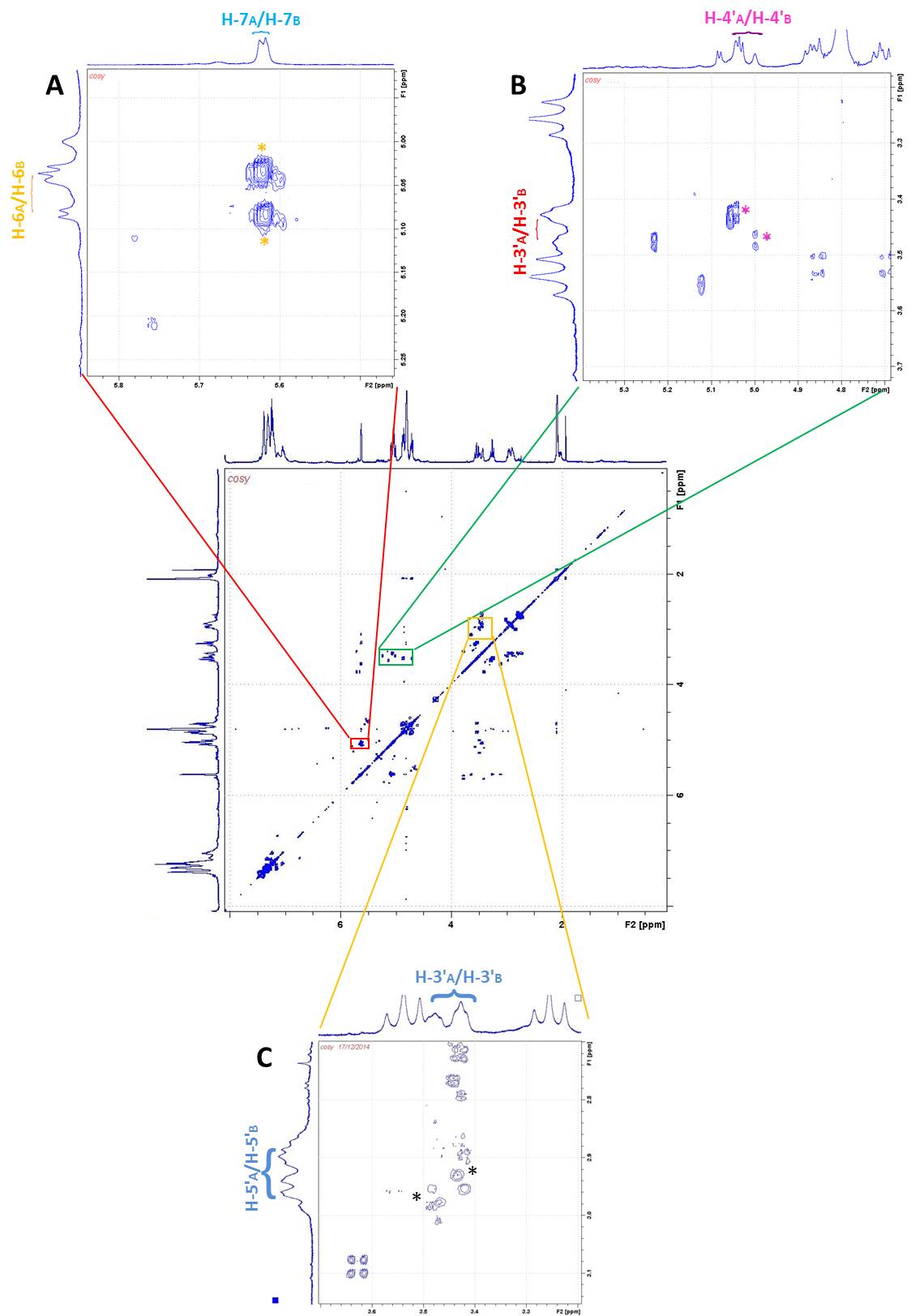

Supplement: S2 Fig — (DOCX) [file pone.0181563.s005.docx]

**S3 Figure: 13C-NMR spectrum of compound 8** (151 MHz, D2O, 298 K).

**
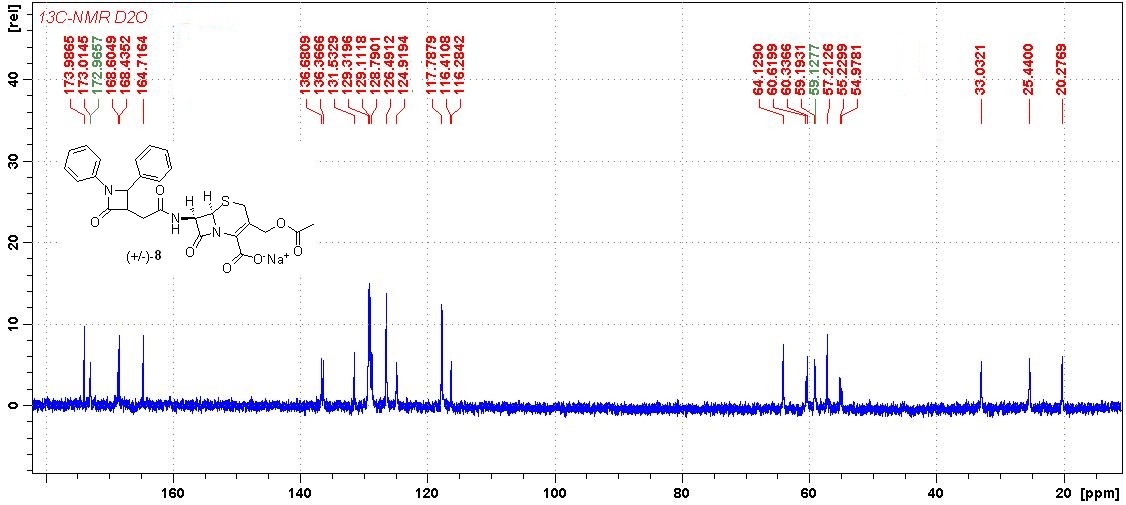
**

Supplement: S3 Fig — (DOCX) [file pone.0181563.s006.docx]

**S4 Figure.** HMQC spectrum of compound **8** (600 MHz, D2O, 298 K)


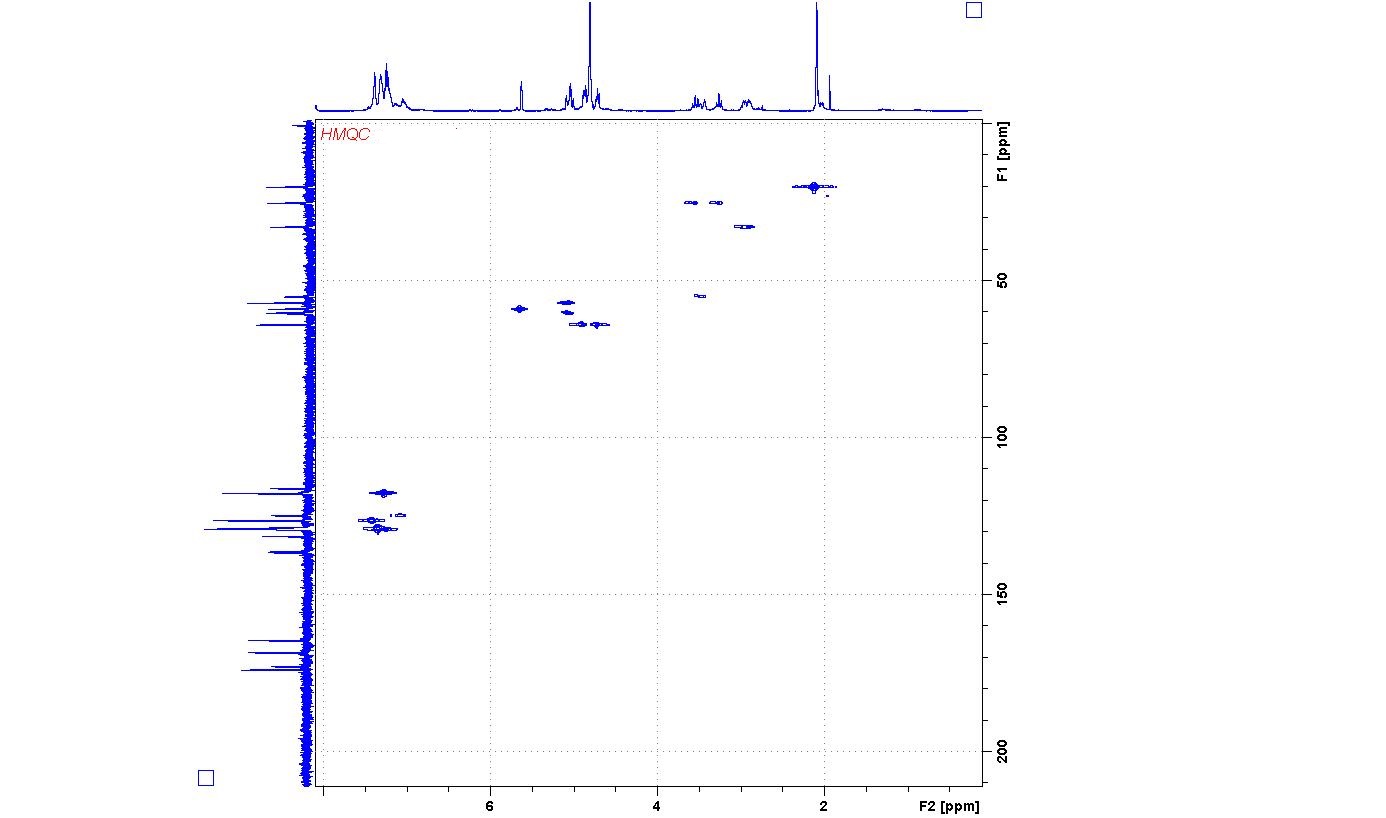

Supplement: S4 Fig — (DOCX) [file pone.0181563.s007.docx]
