# Supplementary material for: Synthesis and biological evaluation of the progenitor of a new class of cephalosporin analogues, with a particular focus on structure-based computational analysis
Source: PLoS One. 2017 Jul 27;12(7):e0181563. doi: 10.1371/journal.pone.0181563 (PMC5531512; doi:10.1371/journal.pone.0181563)
Supplement: S5 Fig — Superposition of the best result obtained with the self-docking procedure on selected complexes, and the crystallographic complex, for: A) 2EX8; b) 3OCL; c) 3VSL. The ligand is represented in sticks. Red: crystallographic conformation. Blue: best pose obtained by the covalent docking strategy. The picture has been prepared using PyMol Molecular Graphics System, v. 1.7.4. (Schrödinger, LLC). (DOCX) [file pone.0181563.s008.docx]

**S5 Figure:** superposition of the best result obtained with the self-docking procedure on selected complexes, and the crystallographic complex, for: A) 2EX8; b) 3OCL; c) 3VSL. The ligand is represented in sticks. Red: crystallographic conformation. Blue: best pose obtained by the covalent docking strategy. The picture has been prepared using PyMol Molecular Graphics System, v. 1.7.4. (Schrödinger, LLC).

**
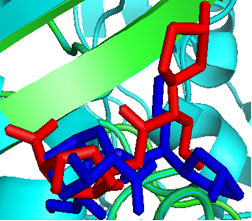

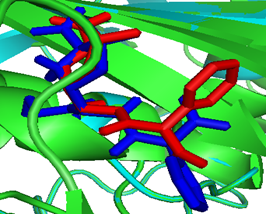

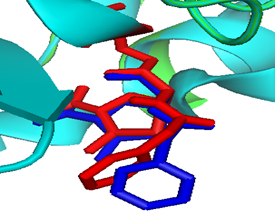
A B C**
